# Multi-omics study identifies novel signatures of DNA/RNA, amino acid, peptide, and lipid metabolism by simulated diabetes on coronary endothelial cells

**DOI:** 10.1038/s41598-022-16300-5

**Published:** 2022-07-14

**Authors:** Aldo Moreno-Ulloa, Hilda Carolina Delgado-De la Herrán, Carolina Álvarez-Delgado, Omar Mendoza-Porras, Rommel A. Carballo-Castañeda, Luis Donis-Maturano, Francisco Villarreal

**Affiliations:** 1 MS2 Laboratory, Biomedical Innovation Department, CICESE, Carretera Ensenada-Tijuana No. 3918, Zona Playitas, 22860 Ensenada, Baja California Mexico; 2 Specialized Laboratory in Metabolomics and Proteomics (MetPro), CICESE, Ensenada, Baja California, Mexico; 3 Posgrado en Ciencias de la Vida, CICESE, Ensenada, Baja California Mexico; 4 Mitochondrial Biology Laboratory, Biomedical Innovation Department, CICESE, Ensenada, Baja California Mexico; 5 CSIRO Livestock and Aquaculture, Queensland Bioscience Precinct, 306 Carmody Rd, St Lucia, QLD Australia; 6 Facultad de Estudios Superiores (FES)-Iztacala, UNAM, Mexico City, Mexico; 7 School of Medicine, University of California, San Diego, CA USA; 8 San Diego VA Healthcare System, San Diego, CA USA

**Keywords:** Proteomics, Metabolomics, Peptides, Proteomics, Mechanisms of disease, Cellular signalling networks

## Abstract

Coronary artery endothelial cells (CAEC) exert an important role in the development of cardiovascular disease. Dysfunction of CAEC is associated with cardiovascular disease in subjects with type 2 diabetes mellitus (T2DM). However, comprehensive studies of the effects that a diabetic environment exerts on this cellular type are scarce. The present study characterized the molecular perturbations occurring on cultured bovine CAEC subjected to a prolonged diabetic environment (high glucose and high insulin). Changes at the metabolite and peptide level were assessed by Liquid Chromatography–Mass Spectrometry (LC–MS^2^) and chemoinformatics. The results were integrated with published LC–MS^2^-based quantitative proteomics on the same in vitro model. Our findings were consistent with reports on other endothelial cell types and identified novel signatures of DNA/RNA, amino acid, peptide, and lipid metabolism in cells under a diabetic environment. Manual data inspection revealed disturbances on tryptophan catabolism and biosynthesis of phenylalanine-based, glutathione-based, and proline-based peptide metabolites. Fluorescence microscopy detected an increase in binucleation in cells under treatment that also occurred when human CAEC were used. This multi-omics study identified particular molecular perturbations in an induced diabetic environment that could help unravel the mechanisms underlying the development of cardiovascular disease in subjects with T2DM.

## Introduction

Damage to coronary artery endothelial cells (CAEC) leads to coronary endothelial dysfunction, which is associated with the development of cardiovascular disease (CVD) in subjects with and without coronary atherosclerosis^1^. Subjects with type 2 diabetes mellitus (T2DM) are particularly at increased risk of myocardial infarction^2^ and coronary endothelial dysfunction has been implicated in the prognosis^3^. A high-glucose (HG) environment—a hallmark of T2DM—leads to nitric oxide signaling, cell cycle^4^, apoptosis^5^, angiogenesis^6^, and DNA structure impairment^7^. However, given the intrinsic heterogeneity of the endothelium, the molecular perturbations caused by HG vary accordingly with the type of studied endothelial cells^8,9^. For instance, human microvascular endothelial cells showed increased gene expression of endothelial nitric oxide synthase, superoxide dismutase 1, glutathione peroxidase 1, thioredoxin reductase 1 and 2 compared to the regulation observed in human umbilical vein endothelial cells (HUVEC) when cultured in HG for 24 h. Furthermore, the response of endothelial cells to HG is influenced by the duration of exposure^10,11^ as demonstrated in bovine aortic and human microvascular endothelial cells where cell proliferation and apoptosis were higher at < 48 h compared to 8 weeks of exposure^10^. In another example of time-dependent response, increased apoptosis (derived from DNA fragmentation) and tumor necrosis factor alpha protein levels were reported in human coronary artery endothelial cells (HCAEC) after only 24 h of incubation with HG^5^. Hence, the molecular response to HG cannot be generalized among endothelial cell types.

Incubation periods up to 48 h are commonly used to evaluate the molecular perturbations caused by HG on endothelial cells wherein transient increases in cell proliferation were shown. This reflects early cell adaptations to the environment rather than cell injury^10,12^. Yet, other studies reported impaired mitochondrial function/structure and nitric oxide signaling in HG treated HCAEC for 48 h^13^. A 72-h study documented an increase in pro-inflammatory cytokines^14^ and oxidative stress in HCAEC^15^. However, the long-term (e.g., > 72 h) effect of HG in CAEC has not been as extensively documented compared to other endothelial cell types (i.e., HUVEC, Human Aortic Endothelial Cells).

Characterizing the long-term effects of HG on CAEC, cells intrinsically involved in the development of CVD, may allow us to better identify key signaling pathways (or specific biomolecules) associated with the development of endothelial dysfunction and CVD.

Here, we test the hypothesis that prolonged simulated diabetes impairs multiple signaling pathways or signal transductions in CAEC. To achieve that, we performed a Liquid Chromatography–Mass Spectrometry (LC–MS^2^)-based multi-omics study utilizing various chemoinformatic and bioinformatic pipelines to extract biological data at a metabolomic, peptidomic, and proteomic level in CAEC perturbed by simulated diabetes. Molecular responses to simulated diabetes hinted at changes in the endothelial phenotype validated by fluorescence microscopy analysis. Our methodological approach facilitated the identification of signaling pathways and specific molecules associated with the development of coronary artery endothelial dysfunction and CVD.

## Methods

### Chemical and reagents

Recombinant human insulin was purchased from Sigma Aldrich (St. Louis, MO, USA). Antibiotic-antimitotic solution, trypsin–EDTA solution 0.25%, Hank’s Balanced Salt Solution (HBSS) without phenol red, Dulbecco’s Modified Eagle’s Media (DMEM) with glutamine, Fetal Bovine Serum (FBS), Hoechst 33258, Pentahydrate (bis-Benzimide)-FluoroPure, MitoTracker Red FM (MTR) and methanol-free formaldehyde (16% solution) were obtained from Thermo Fisher Scientific (Waltham, MA, USA). Methanol, Acetonitrile, and water were Optim LC–MS Grade and obtained from Fisher Scientific (Hampton, NH, USA). Ethanol LiChrosolv Grade was obtained from Merck KGaA (Darmstadt, Germany). Rabbit anti-Von Willebrand factor (vWf) antibody and goat anti-rabbit IgG conjugated to Alexa Fluor 488 were obtained from Abcam (Cambridge, MA, USA).

### Cell culture

BCAEC were purchased from Cell applications, Inc. (San Diego, CA, USA) and grown as previously described^16^. In brief, cells were proliferated in 100 mm × 20 mm dishes with DMEM (5.5 mmol/L glucose, supplemented with 10% FBS and 1% antibiotic-antimitotic solution) or growth-media at 37 °C in an incubator with a humidified atmosphere of 5% CO_2_.

### Cellular treatment

Cells were seeded at 300,000 cells per well in 6-well plates (Corning CellBIND) and allowed to proliferate (using growth-media) until reaching an 80% confluence. Then, cells were switched to DMEM with 1% FBS for 12 h to maintain the cells under a quiescent state. Cells were first treated with 100 nmol/L insulin (high-insulin, HI) or vehicle in normal glucose (NG, 5.5 mmol/L in DMEM) for 3 days^17^ and then different concentrations of glucose were added to the media to achieve a high-glucose (HG: 15 mmol/L, 17.2 mmol/L or 20 mmol/L) ambient for up to 9 days. This sequential scheme tried to mimic the pathophysiological conditions that occur in T2DM patients, wherein hyperinsulinemia precedes hyperglycemia^18^. The control or NG group consisted of glucose 5.5 mmol/L without HI. As hyperosmolar controls, cells were incubated with 5.5 mmol/L glucose + mannitol 9.5, 12.2 and 14.5 mmol/L with or without HI for 9 days. For selected experiments (binucleation analysis), HCAEC (55 years old Caucasian male, history of T2DM for > 5 years) were purchased from Cell Applications, Inc. and subjected to the same conditions as BCAEC but using MesoEndo Growth Medium (Cell Applications, Inc.) to induce proliferation. For simulated diabetes, HCAEC were treated with HI and HG (20 mmol/L) for 9 days as with BCAEC but, MesoEndo Growth Medium was used instead. For all the experiments, three independent wells (in either 6 or 12 wells-plate) per condition were pooled to generate an independent biological replicate. BCAEC and HCAEC were used at passages between 6 and 12.

### Measurement of mitochondrial membrane potential by flow cytometry

To evaluate the individual and combined metabolic effects of HI and HG on BCAEC, we measured the mitochondrial membrane potential, as a surrogate marker of oxidative metabolism^19^, by flow cytometry. After treatment (in 12-wells plate), BCAEC were gently collected using a sterile cell scraper, washed with pre-warmed HBSS, and centrifuged to obtain a cell pellet. Cells were re suspended in fresh pre-warmed HBSS and incubated with 100 nmol/L MTR for 30 min at 37 °C under a dark environment. Cells were washed 3 × with HBSS (to remove the excess of dye), and finally re suspended in fresh HBSS for data acquisition. Flow cytometry analysis was performed using an Attune Acoustic Focusing Flow Cytometer (Thermo Fisher Scientific, Waltham, MA, USA). A minimum of 10,000 cells was recorded and data was processed using FlowJo (v10.6.1, Becton, Dickinson and Company, Franklin Lakes, NJ, USA).

### Immunofluorescence

As previously described^16^, 100,000 cells per well were seeded onto 12-well plates (Corning CellBIND) and exposed to simulated diabetes. Thereafter, BCAEC and HCAEC were washed with PBS to remove dead cells and debris. Cells were fixed, permeabilized, and blocked as described before^20^. Cells were then incubated with a polyclonal antibody against the vWf (1:400, 3% BSA in PBS) overnight at 4 °C and thereafter washed 3 × with PBS. Alexa Fluor 488-labeled anti-rabbit (1:400 in PBS) was then used as a secondary antibody for 1 h at RT and washed 3 × with PBS. As a negative control, cells were incubated only with secondary antibody to assess for non-specific binding. Cell nuclei were stained with Hoechst 33258 (2 µg/mL in HBSS) for 30 min and washed 3 × with PBS. Fluorescent images were taken in at least three random fields per condition using an EVOS FLoid Cell Imaging Station with a fixed 20 × air objective. Image analysis was performed through ImageJ software (version 2.0.0).

### Peptide and non-peptide metabolite extraction

**Figure 1:**
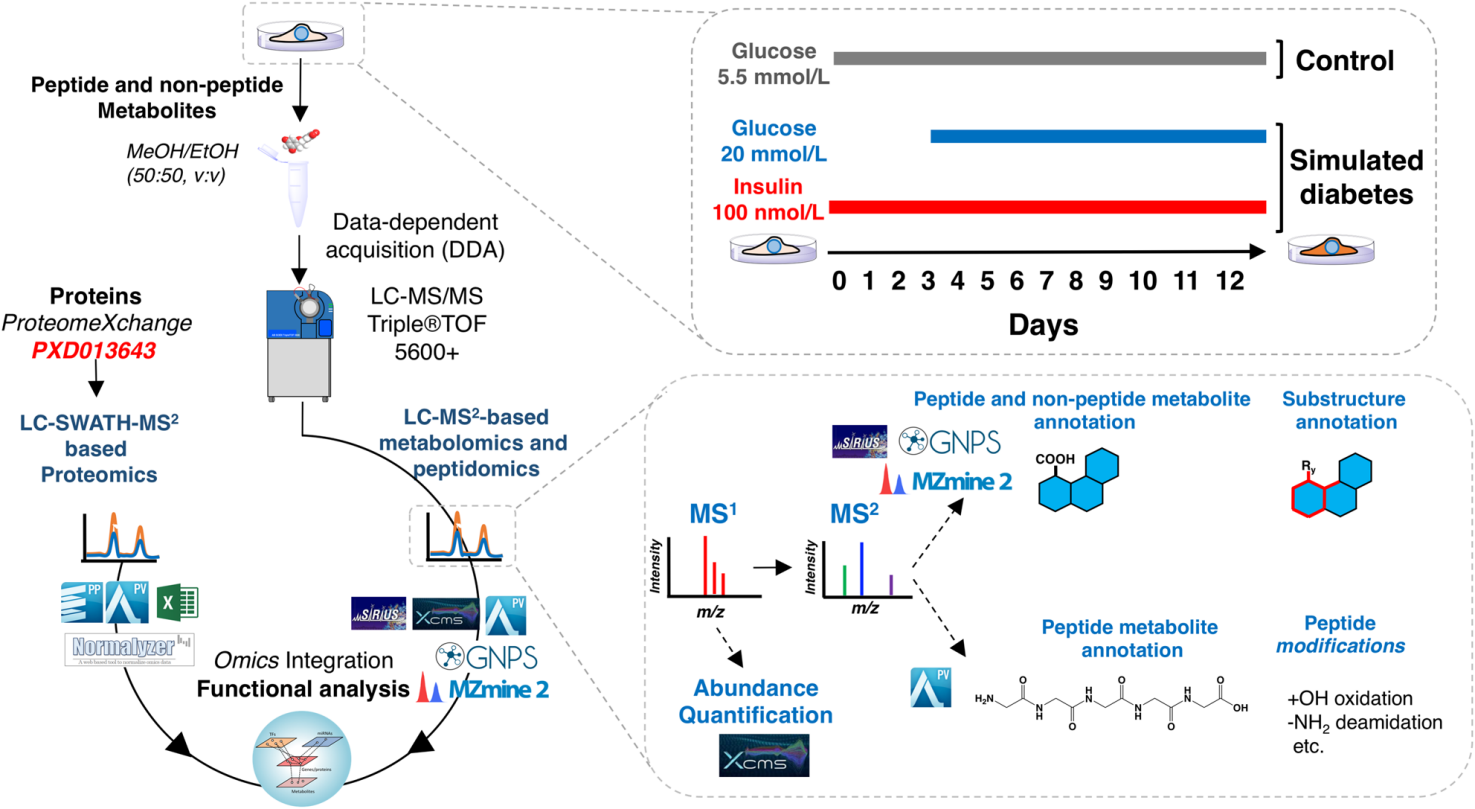
Illustration of the methodology followed in this study.

Cells were seeded at 300,000 cells per well in 6-well plates (Corning CellBIND) and treated as above. After HG and HI conditions, metabolites were extracted following a published protocol for adherent cells with some modifications^21^ (Fig. 1).

In brief, after washing the cells 3 × with PBS, 500 µL of a cold mixture of methanol: ethanol (50:50, v:v) were added to each well, covered with aluminum foil, and incubated at − 80 °C for 4 h. Cells were then scrapped using a lifter (Fisher Scientific, Hampton, NH, USA), and the suspension was transferred to Eppendorf tubes before centrifugation for 10 min at 14,000 rpm at 4 °C. The supernatant was transferred to another tube and dried down by SpeedVac System (Thermo Fisher Scientific, Waltham, MA, USA). Samples were reconstituted in water/acetonitrile 95:5 v/v with 0.1% formic, centrifuged at 14,000 rpm for 10 min at 4 °C. The particle free supernatant was recovered for further LC–MS^2^ analysis.

### LC–MS^2^ data acquisition for untargeted metabolomics and peptidomics

Metabolites were loaded into an Eksigent nanoLC 400 system (AB Sciex, Foster City, CA, USA) with a HALO Phenyl-Hexyl column (0.5 × 50 mm, 2.7 μm, 90 Å pore size, Eksigent AB Sciex, Foster City, CA, USA) for data acquisition using the LC–MS parameters previously described with some modifications^22^. In brief, the separation of metabolites was performed using gradient elution with 0.1% formic acid in water (A) and 0.1% formic acid in ACN (B) as mobile phases at a constant flow rate of 5 μL/min. The gradient started with 5% B for 1 min followed by a stepped increase to 100%, B over 26 min and held constant for 4 min. Solvent composition was returned to 5% B for 0.1 min. Column re-equilibration was carried out with 5% mobile phase B for 4 min. Potential carryover was minimized with a blank run (1 μL buffer A) between sample experimental samples. The eluate from the LC was delivered directly to the TurboV source of a TripleTOF 5600 + mass spectrometer (AB Sciex, Foster City, CA, USA) using electrospray ionization (ESI) under positive mode. ESI source conditions were set as follows: IonSpray Voltage Floating, 5500 V; Source temperature, 350 °C; Curtain gas, 20 psi; Ion source gases 1 and 2 were set to 40 and 45 psi; Declustering potential, 100 V. Data was acquired using data-dependent acquisition (DDA) with high sensitivity mode selected, automatically switching between full-scan MS and MS/MS. The accumulation time for TOF MS was 0.25 s/spectra over the *m/z* range 100–1500 Da and for MS/MS scan was 0.05 s/spectra over the *m/z* 50–1500 Da. The DDA settings were as follows: charge state + 1 to + 2, intensity 125 cps, exclude isotopes within 6 Da, mass tolerance 50 mDa, and a maximum number of candidate ions 20. Under IDA settings, the ‘‘exclude former target ions’’ was set as 15 s after two occurrences and ‘‘dynamic background subtract’’ was selected. Manufacturer rolling collision energy (CE) option was used based on the size and charge of the precursor ion using formula CE = *m/z* × 0.0575 + 9. The instrument was automatically calibrated by the batch mode using appropriate positive TOF MS and MS/MS calibration solutions before sample injection and after injection of two samples (< 3.5 working hours) to ensure a mass accuracy of < 5 ppm for both MS and MS/MS data. Instrument performance was monitored during data acquisition by including QC samples (pooled samples of equal volume) every 4 experimental samples. Data acquisition of experimental samples was also randomized.

### Processing of LC–MS^2^ for untargeted metabolomics

To identify peptide and non-peptide metabolites (hereafter referred as to untargeted metabolomics), we followed a workflow comprised of open-access software packages and on-line platforms (for library spectral matching) commonly used for untargeted metabolomics. Three complementary informatic approaches were utilized to analyze the LC–MS^2^ datasets: (1) feature extraction, alignment, normalization, and univariate statistical analysis was performed using the XCMS (version 2.7.2) online platform (https://xcmsonline.scripps.edu)^23^; (2) MS^2^ spectral data extraction for metabolite identification (Metabolomics Standards Initiative (MSI) classification level 2 and 3)^24^ was performed with MZmine (version 2.53)^25^, the Global Natural Products Social Molecular Networking web platform (GNPS, https://gnps.ucsd.edu)^26,27^, and in-silico integrated tools within SIRIUS software (version 4.9.12)^28^, and; (3) multivariate statistical analysis and heatmap visualization was done using Metaboanalyst 5.0 (https://www.metaboanalyst.ca)^29^. For approach 1, raw proprietary .wiff files were uploaded into the XCMS online platform to perform mass detection, chromatogram building and deconvolution, isotopic assignment, feature alignment, and gap-filling (to detect features missed during the initial alignment). For approach 2, .wiff files were first converted to .mzML using ProteoWizard version 3.0 and then imported into MZmine to perform the peak extraction steps as in the XCMS online platform. To identify or annotate the metabolites at the chemical structure and class level, the MS^2^-containing features extracted with MZmine were further analyzed by molecular networking using the GNPS platform and associated in-silico dereplication tools, Network Annotation Propagation (NAP)^30^ and MS2LDA^31^, as well as the automated chemical classification by Classyfire^32^, as previously described^22^. The confidences of such annotations are level 2 (probable structure by library spectrum match) and level 3 (tentative candidates) in agreement with the Metabolomics Standards Initiative (MSI) classification^24^. Molecular networking, NAP, and Classyfire outputs were integrated using the MolNetEnhancer workflow^33^. Molecular networks were visualized using Cytoscape version 3.8.2^34^. In addition, chemical substructures (co-occurring fragments and neutral losses referred to as “mass2motifs” [M2M]) were recognized using the MS2LDA web pipeline (http://www.ms2lda.org) to further annotate metabolites (level 3, MSI). For select metabolites, high-confidence annotations (at the substructure and structure pattern, level 3, MSI) were made using in-silico integrated tools within SIRIUS software (version 4.9.12)^28^. For approach 3, normalized peak abundance data (.txt) retrieved by XCMS analysis was imported into Metaboanalyst and filtered for Principal Component Analysis (PCA) and HeatMap analysis. The detailed processing parameters for all the pipelines are found in supplemental experimental methods in supporting information.

### Processing of LC–MS^2^ for peptidomics

To expand the identification of endogenous peptide metabolites not contained in standards-enriched spectral libraries (including GNPS and collaborators), we performed in-silico peptide identification using the Bos taurus proteome as a database to retrieve amino acid sequences for spectral matching (hereafter referred as to peptidomics). The raw files (derived from the same experiments) used for untargeted metabolomics (.wiff and .wiff.scan files) from the experimental and control groups were analyzed separately using ProteinPilot software version 4.2 (Ab Sciex, Foster City, CA, USA) with the Paragon algorithm. MS^1^ and MS^2^ data were searched against the *Bos taurus* SwissProt sequence database (6006 reviewed proteins + common protein contaminants, February 2019 release). The parameters input was: sample type, identification; digestion, none; Cys alkylation, none; instrument, TripleTOF 5600; special factors, none; species, *Bos taurus*; ID focus, biological modifications, and amino acid substitutions; search effort, thorough ID. False discovery rate analysis was also performed. All peptides were exported and those with a > 90% confidence were linked to the corresponding feature extracted by the XCMS algorithm using their accurate mass and retention time information. For peptide quantification, we employed the normalized feature abundances (MS^1^ level) generated by XCMS. A significance threshold of *p* < *0.05* (Welch’s t test) was utilized.

### Processing of LC–SWATH–MS^2^ for proteomics

The SWATH-based proteomics data (identifier PXD013643), hosted in ProteomeXchange consortium via PRIDE^35^, was reanalyzed with some modifications. This dataset derived from independent biological replicates as those used in this study. The parameters used to build the spectral library remained the same^16^, while the parameter for peptides per protein was set to 100 in the software SWATH Acquisition MicroApp 2.0 in PeakView version 1.2 (AB Sciex, Foster City, CA, USA). The obtained protein peak areas were exported to Markerview version 1.3 (AB Sciex, Foster City, CA, USA) for further data refinement, including assignment of IDs to files and removal of reversed and common contaminants. Peak areas were exported in a .tsv file, and normalized with NormalyzerDE online version 1.3.4^36^. The NormalyzerDE pipeline comprises 8 different normalization methods (Log2, variance stabilizing normalization, total intensity, median, mean, quantile, CycLoess, and robust linear regression). The results of qualitative (MA plots, scatter plots, box plots, density plots) and quantitative (pooled intragroup coefficient of variation [PCV], median absolute deviation [PMAD], estimate of variance [PEV]) parameters were compared between the normalization methods to select the most appropriate.

### Bioinformatic analysis of proteomics data

Proteins that passed the significance threshold were first converted to their corresponding Entrez Gene (GeneID) using https://www.uniprot.org/uploadlists/ and then transformed to their human equivalents using the ortholog conversion feature in https://biodbnet-abcc.ncifcrf.gov/db/dbOrtho.php. Bioinformatic analysis was done on OmicsNet website platform (https://www.omicsnet.ca/)^37,38^. First, a protein–protein interaction (PPI) molecular network (first-order network containing query or seeds molecules and their immediate interacting partners) with minimum network filtering selected using STRING PPI database was built^39^, and then pathway enrichment analysis was performed using the built-in REACTOME^40^ and the Kyoto Encyclopedia of Genes and Genomes (KEGG)^41^ databases. Hypergeometric test was used to compute *p* values.

### Integrative bioinformatic analysis of proteomics and metabolomics data

The molecular interactions between the proteins and metabolites differentially abundant between HG + HI and NG were determined in OmicsNet. The lists of proteins (EntrezGene ID) and metabolites (HMDB ID) were loaded to build a composite network using protein–protein (STRING database selected) and metabolite-protein (KEGG database selected) interaction types. The primary network relied on the metabolite input using the minimum network filtering option. Pathway enrichment analysis was performed using the built-in REACTOME and KEGG databases. Hypergeometric test was used to compute *p* values.

### Statistical analysis

All experiments were performed with biological triplicates. Three independent wells per condition were pooled to generate an independent biological replicate. For all the data, excluding proteomics and metabolomics, statistical analysis was performed by either using one-way ANOVA followed by the Dunnett’s post hoc test or unpaired student’s t test, as appropriate. A *p* value ≤ 0.05 was considered statistically significant. For the proteomics data, based on the high reproducibility of SWATH-based quantification^42,43^ and fold-change compression phenomenon (i.e., lower measured fold-change vs. real biological fold-change)^43,44^, proteins with a fold change ≥ 1.2 or ≤ 1/1.2 and a *p* value < 0.05 (Welch’s t-test) were considered as differentially abundant between NG and HG + HI conditions. For the metabolomics data (DDA), also taking into account the fold-change compression phenomenon^44^—but lower accuracy of DDA than DIA-SWATH, features with a fold change ≥ 1.3 or ≤ 1/1.3 and a *p* value < 0.05 (Welch’s t-test) were considered as differentially abundant. We did not apply multiple-test corrections to calculate adjusted *p* values, because this process could obscure proteins or metabolites with real changes (true-positives)^45^. Instead, the analysis was focused on top-enriched signaling pathways (adjusted *p* value < 0.01) that allowed us to determine a set of interacting proteins and metabolites with relevant biological information and contributes in reducing false positives. For multivariate statistical analysis and heatmap visualization, Metaboanalyst 4.0 (https://www.metaboanalyst.ca) was utilized. PCA on log-transformed data was used to assess for sample clustering behavior and inter-group variation. No scaling was used for PCA and heatmap analysis. Software PRISM 6.0 (GraphPad Software, San Diego, CA) was used for the creation of volcano plots and column graphs.

## Results

### Mitochondrial membrane potential

To define a simulated diabetes model, we tested the effects (time and concentration-dependent) of various HG concentrations with and without a single concentration of HI (100 nmol/L) on the endothelial mitochondrial function^19^. We noted differential effects of HG depending on the incubation time (3 vs. 6 vs. 9 days) and the presence of HI (Fig. S1). Our results showed that 20 mmol/L glucose with 100 nmol/L insulin at day 9 significantly reduced the mitochondrial membrane potential (Fig. S1). Hyperosmolar controls at day 9 (using mannitol) did not significantly affect the mitochondrial endpoint. Therefore, our simulated diabetes model was defined as an environment of 20 mmol/L glucose + 100 nmol/L insulin for 9 days, while the control groups consisted of cells treated with 5.5 mmol/L glucose for the same period.

### Untargeted metabolomics

**Figure 2:**
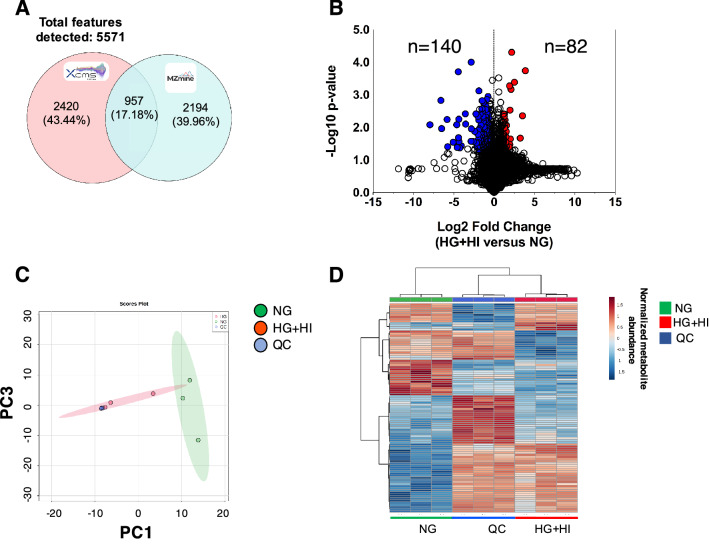
Simulated diabetes induced changes in the metabolome of bovine coronary artery endothelial cells (BCAEC). (**A**) Venn diagram of features identified among MZmine and XCMS software (0.01 Da and 1 min retention time, thresholds) on LC–MS^2^ datasets. (**B**) Volcano plot of all quantified metabolites displaying differences in relative abundance (> ± 30% change, < 0.05 *p* value cut-offs) between BCAEC cultured in control (NG) media and simulated diabetes (HG + HI) for twelve days. Values (dots) represent the HG + HI/NG ratio for all metabolites. Red and blue dots denote downregulated and upregulated metabolites in the HG + HI group versus NG group, respectively. (**C**) Principal Component Analysis (PCA) of LC–MS^2^ datasets. Data was log transformed without scaling. Shade areas depict the 95% confidence intervals. (**D**) HeatMap of the top 100 metabolites ranked by t-test. Abbreviations: *NG* normal glucose, *HG* high glucose, *HI* high insulin, *QC* quality control.

Overall 5571 features or potential metabolites were detected using XCMS and MZmine, wherein 957 (~ 18%) features were commonly identified in both platforms (Fig. 2A). Based on the relative quantification using XCMS, 140 and 82 features were detected with reduced and increased abundances respectively in the experimental group compared to the control group (Fig. 2B). The effects of HG and HI in the experimental group are observed by PCA analysis wherein the experimental samples clustered away from the control group (Fig. 2C). The consistency of the LC–MS equipment is apparent by the tight-clustering of the QC samples (Fig. 2C). Further, the heatmap visualization of the top 100-modulated metabolites exhibited the different distribution patterns among groups (Fig. 2D). Using the GNPS platform for automatic metabolite annotation, 106 compounds (excluding duplicates and contaminants) were putatively annotated with a level 2 confidence annotation (MS^2^ spectral match) (Table S1) in agreeance with the MSI classification^24^. Some metabolites identified by the GNPS platform could not be quantified because they were not detected by the XCMS algorithm during feature area normalization and quantification.

**Figure 3:**
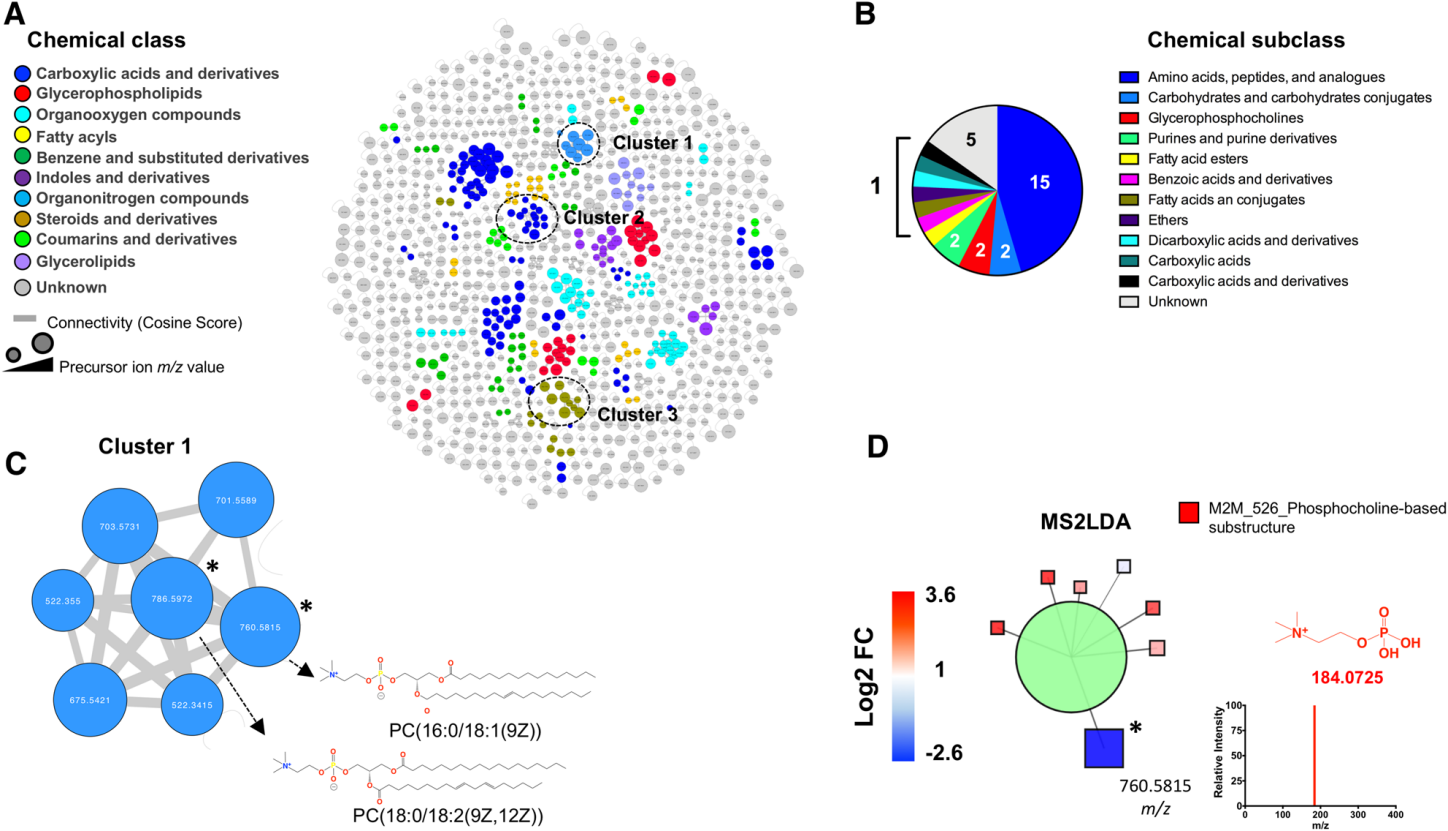
Bovine coronary artery endothelial cells (BCAEC) metabolite molecular network. (**A**) Molecular classes (according to Classyfire) of the metabolome identified by the MolNetEnhancer workflow and visualized by Cytoscape version 3.8.2. Each node represents a unique feature and the color of the node denotes the associated chemical class. The thickness of the edge (connectivity) indicates the MS^2^ similarity (Cosine score) among features. The *m/z* value of the feature is shown inside the node and is proportional to the size of the node. Three selected clusters or connected features as relevant are shown. (**B**) Chemical subclasses (predicted by CANOPUS) of the dysregulated metabolites by simulated diabetes. (**C**) Inset of cluster 1 denoting the presence of phosphocholine (PC)-containing lipids. Significant differential abundant features among simulated diabetes (HG + HI) and control (NG) groups are indicated with an asterisk (*p* value < 0.05). (**D**) Characterization of features in (**B**) aided by substructure recognition by MSLDA software using MS^1^ visualization in www.ms2lda.org. Fragment at *m/z* 184.0725 linked to a PC head group by mzCloud in silico prediction (www.mzCloud.org). Abbreviations: *M2M* mass2motif, *FC* fold change, *NG* normal glucose, *HG* high glucose, *HI* high insulin. Chemical structures were drawn by ChemDraw Professional version 16.0.1.4.

Moreover, GNPS Molecular Networking aligned the MS^2^-containing features (n = 1013) based on their structural similarity, creating 118 independent networks or clusters with at least two connected nodes (Fig. 3A). The use of MolNetEnhancer workflow allowed to putatively identify chemical classes (level 3, MSI) for 56 of the 118 independent networks. The top-10 most abundant annotated chemical classes and associated metabolites are shown in Fig. 3A. A focused analysis on the chemical subclasses associated with the dysregulated metabolites by simulated diabetes, revealed that amino acids, peptides, and analogues were principally affected (Fig. 3B). Three-clusters from the network were further analyzed because they contained annotated metabolites by spectral matching, which facilitates the annotation of other cluster’s nodes. Cluster 1 revealed two metabolites linked to the organonitrogen compounds class with reduced abundance in the experimental group (Fig. 3C). Library spectral match (level 2, MSI) suggest PC(16:0/18:1(9Z)) and PC(18:0/18:2(9Z,12Z)) as putative candidates, which was supported by MS2LDA phosphocholine-substructure recognition (Fig. 3D).

**Figure 4:**
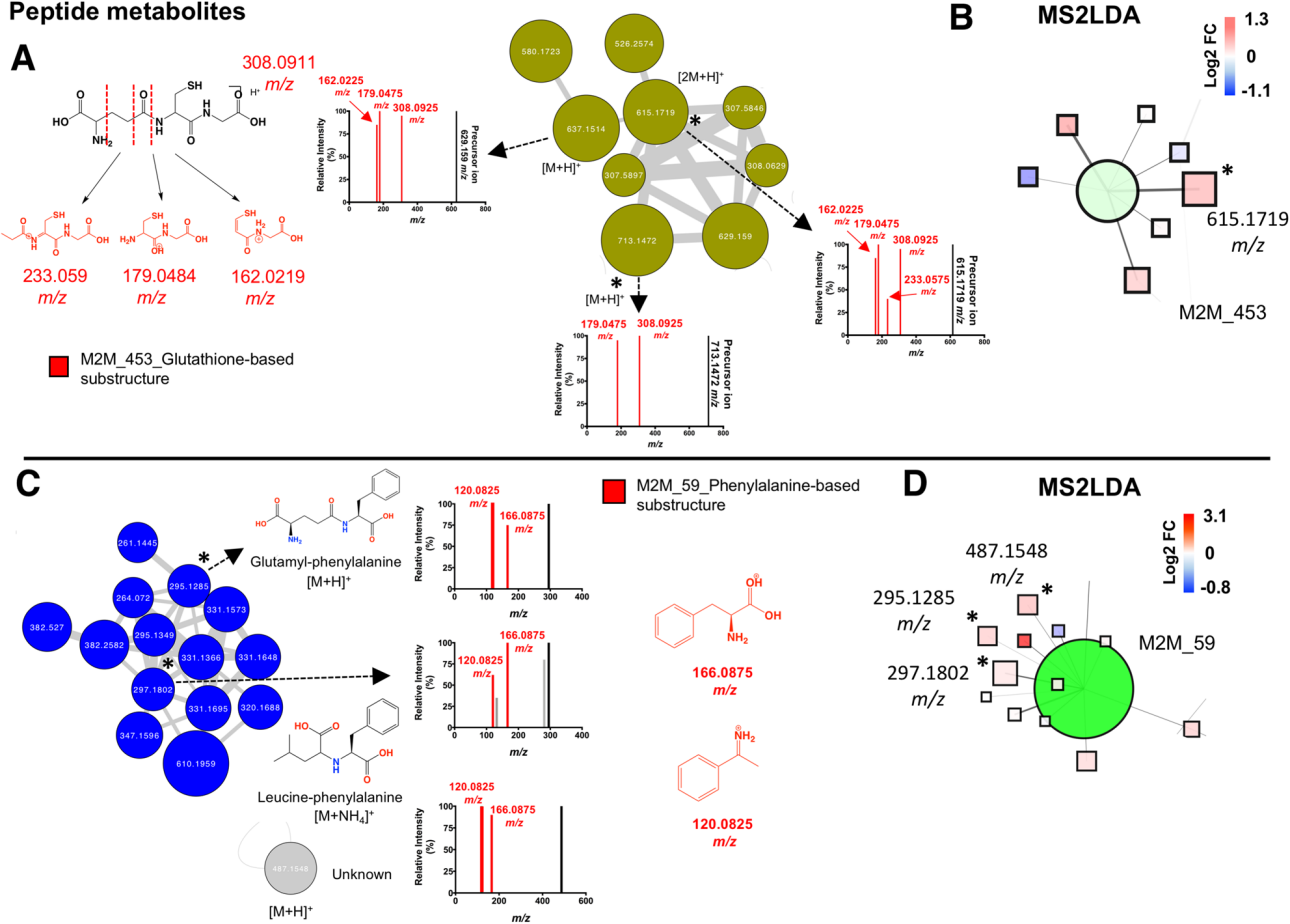
Peptide metabolites modulated by simulated diabetes in bovine coronary artery endothelial cells (BCAEC). (**A**) Cluster 2 retrieved from the main molecular network linked to glutathione and derivatives. The fragments of mass-2-motif (M2M)_453 colored in red are characteristic of a glutathione core and the fragments are shown in red. (**B**) Features associated with M2M_453 using MS^1^ visualization in www.ms2lda.org. (**C**) Cluster 3 retrieved from the main molecular network linked to phenylalanine-based metabolites. A singular node at *m/z* 487.1548 is also shown. The fragments of M2M_59 colored in red are characteristic of a phenylalanine core (Heuristic and Quantum Chemical predictions by www.mzCloud.org). (**D**) Features associated with M2M_59 using MS^1^ visualization in www.ms2lda.org. In GNPS’s clusters (**A** and **C**), the node’s color denotes the chemical class assigned to the cluster. The thickness of the edge (connectivity) indicates the cosine score (MS^2^ similarity). The *m/z* value of the feature is shown inside the node and is proportional to the size of the node. Significant differential abundant features among simulated diabetes (HG + HI) and control (NG) groups are indicated with an asterisk (*p* value < 0.05). In MS2LDA’s nodes (**B** and **D**), the green node represents the M2M and squares indicate individual features. Edges represent connections to M2M. Significant differential abundant features among groups are indicated with an asterisk (*p* value < 0.05). Abbreviations: *M2M* mass2motif, *FC* fold change, *NG* normal glucose, *HG* high glucose, *HI* high insulin. Chemical structures were drawn by ChemDraw Professional version 16.0.1.4.

In cluster 2, glutathione-based metabolites (MSI level 3) were detected through fragments *m/z* 308.0925, 233.0575*,* 179.0475, and 162.0225 retrieved by the M2M_453 substructure and associated with glutathione structure using mzCloud in silico predictions (Fig. 4A). The precursor ion at *m/z* 713.1472 and glutathione (annotated at level 2, MSI) were detected with increased abundance in the experimental group. MS2LDA visualization, at the M2M level, correlated with the GNPS molecular networking clustering (Fig. 4B). In cluster 3, various phenylalanine-based metabolites were putatively annotated aided by MS2LDA substructure recognition (Fig. 4C and D). Within this cluster, glutamyl-phenylalanine, leucine-phenylalanine and the precursor ion at *m/z* 487.1548 (unknown) presented with increased abundance in the experimental versus control group. On the other hand, various amino acids were annotated (level 2, MSI) by GNPS spectral matching and manual inspection of data (Table S2). Threonine, valine, proline, leucine, serine, glutamic acid, methionine, and tyrosine presented increased abundance (fold change range 1.3–1.7, *p* < 0.05) in the experimental versus control group. Particularly, metabolites linked to the catabolism of tryptophan via the serotonin and kynurenine pathway^46^ were annotated (level 2, MSI), including melatonin, acetyl serotonin, and kynurenine (Table S2). However, only kynurenine was significantly elevated in the experimental group. The full list of annotated metabolites, differential abundances and another relevant feature information is shown in Table S2.

### Peptidomics

Experimental and control datasets were analyzed separately to identify the peptides and their biological modifications. The complete list of peptides identified by ProteinPilot between the experimental and control groups are described in Table S3. Proline oxidation was the most frequent biological modification detected in the experimental group datasets. We identified 8 and 12 peptides with a confidence of > 90% in the control and experimental group, respectively. Differential abundance of 2 proline-rich peptides was observed in the experimental group compared to the control group. An additional tripeptide was manually annotated with a LPP sequence (Table S4).

### Proteomics

**Figure 5:**
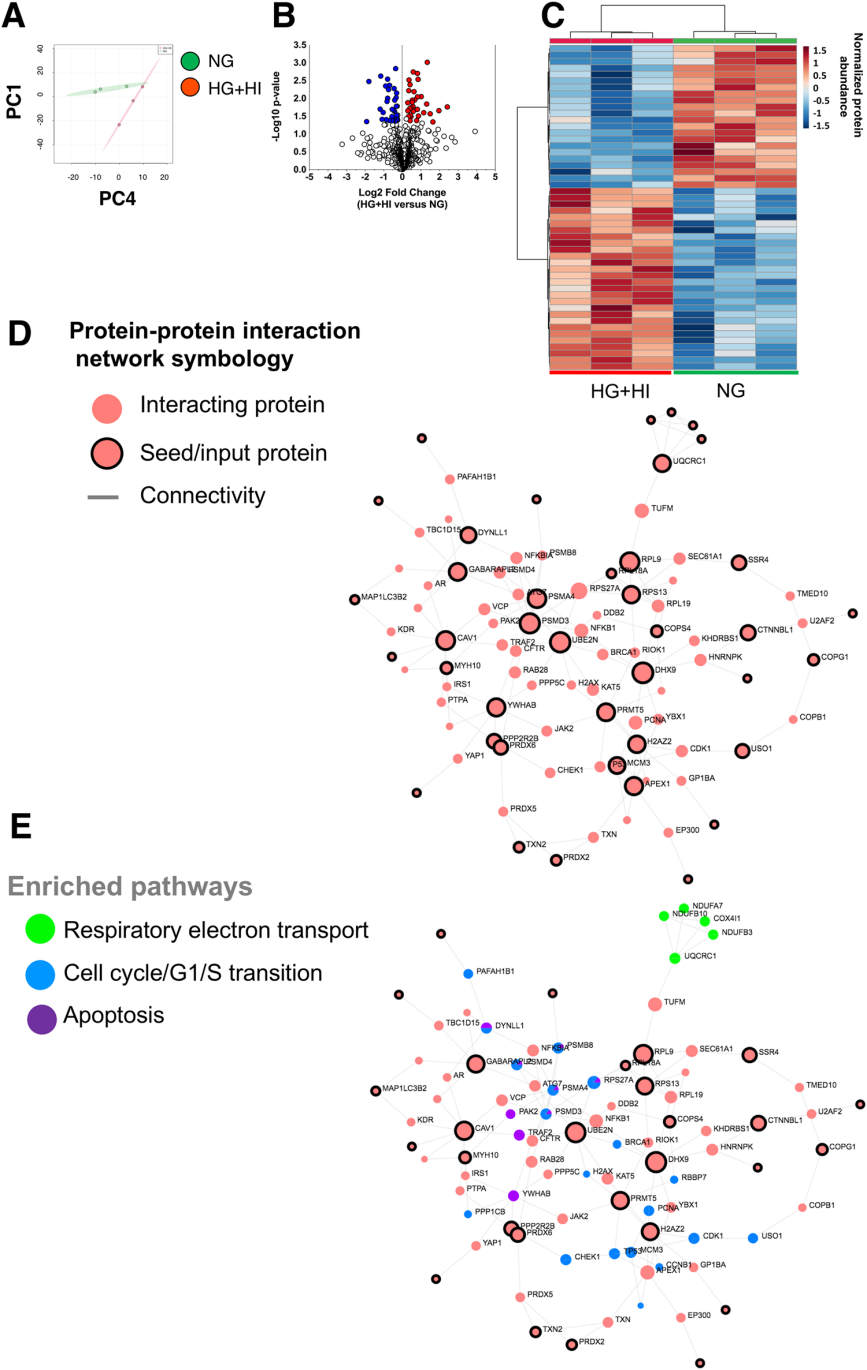
Simulated diabetes induced changes in the proteome of bovine coronary artery endothelial cells (BCAEC). (**A**) Principal Component Analysis (PCA) of LC–SWATH–MS^2^ datasets. Data was log transformed without scaling. Shade areas depict the 95% confidence intervals. No scaling was used. (**B**) Volcano plot of all quantified proteins (Quantile normalization) displaying differences in relative abundance (> ± 20% change, < 0.05 *p* value cut-offs) between BCAEC cultured in control (NG) media and simulated diabetes (HG + HI) for twelve days. Values (dots) represent the HG + HI/NG ratio for all proteins. Red and blue dots denote downregulated and upregulated proteins in the HG + HI group versus NG group, respectively. (**C**) HeatMap of the top 50 proteins ranked by t-test. (**D**) Protein–protein interactome (> 0.9 confidence) generated using the list of the dysregulated proteins by simulated diabetes. Seed or input proteins are illustrated with a black shade and the gene ID is also shown. (E) Select enriched (adjusted *p* value < 0.05) REACTOME pathways^40^ within the protein network. Abbreviations: *NG* normal glucose, *HG* high glucose, *HI* high insulin.

The re-analysis of the SWATH data (PXD013643 dataset) facilitated the identification of 952 quantifiable proteins (717 proteins with at least 2 unique peptides, 1% false discovery rate) and no missing values among technical and biological replicates (Table S5). Sample datasets were normalized using 8 different methods to select the most appropriate based on quantitative and qualitative parameters on our dataset. Quantile normalization produced a better qualitative and quantitative profile and was selected to further process our data (Fig. S1). PCA analysis of normalized data denoted a clear separation of the groups suggesting overall differences in their proteomes (Fig. 5A). Differential abundance analysis revealed 32 and 33 proteins with increased and decreased abundance in the experimental group (Fig. 5B). Further, the heatmap visualization of the top 50-modulated proteins exhibited the different distribution patterns among the experimental and control groups (Fig. 5C). To obtain a molecular insight we performed a functional enrichment analysis using a network-based approach. First, we created a composite network comprising PPI between the modulated proteins by simulated diabetes (seed proteins) and their immediate interacting partners (highest confidence > 0.9) retrieved from STRING Database (incorporated in OmicsNet platform). The principal network using the up-modulated proteins consisted of 91 proteins, 137 edges and 40 seed proteins (nodes with black shadow) and is illustrated in Fig. 5D. The 10 most significant (adjusted *p* value < 0.05) REACTOME and KEGG pathways on the global network are shown in Table 1. Generally speaking, signaling pathways linked to DNA/RNA metabolism, mitochondria and apoptosis were significantly enriched within the PPI network (Fig. 5E).

**Table 1:** Pathway enrichment analysis of the dysregulated proteins by simulated diabetes.

REACTOME database	Total	Hits	FDR	KEGG database	Total	Hits	FDR
Cell cycle	508	19	0.000192	Maturity onset diabetes of the young	149	9	0.00248
Cell cycle checkpoints	131	10	0.000259	Renal cell carcinoma	201	10	0.00248
G1/S transition	113	9	0.000489	SNARE interactions in vesicular transport	124	8	0.00248
G1/S DNA Damage checkpoints	62	7	0.000606	Transcriptional misregulation in cancer	186	9	0.00549
Activation of NF-kappaB in B cells	66	7	0.000671	Human T-cell leukemia virus 1 infection	162	8	0.00998
Synthesis of DNA	95	8	0.000671	Phagosome	65	5	0.018
DNA replication	102	8	0.000824	Aldosterone synthesis and secretion	69	5	0.0206
Regulation of activated PAK-2p34 by proteasome mediated degradation	48	6	0.000824	Mitophagy—animal	72	5	0.0206
Mitotic G1-G1/S phases	140	9	0.000824	Chemical carcinogenesis	201	8	0.0206
APC/C:Cdc20 mediated degradation of mitotic proteins	76	7	0.000824	Human papillomavirus infection	155	7	0.0206

### Integration of metabolomics and proteomics

**Figure 6:**
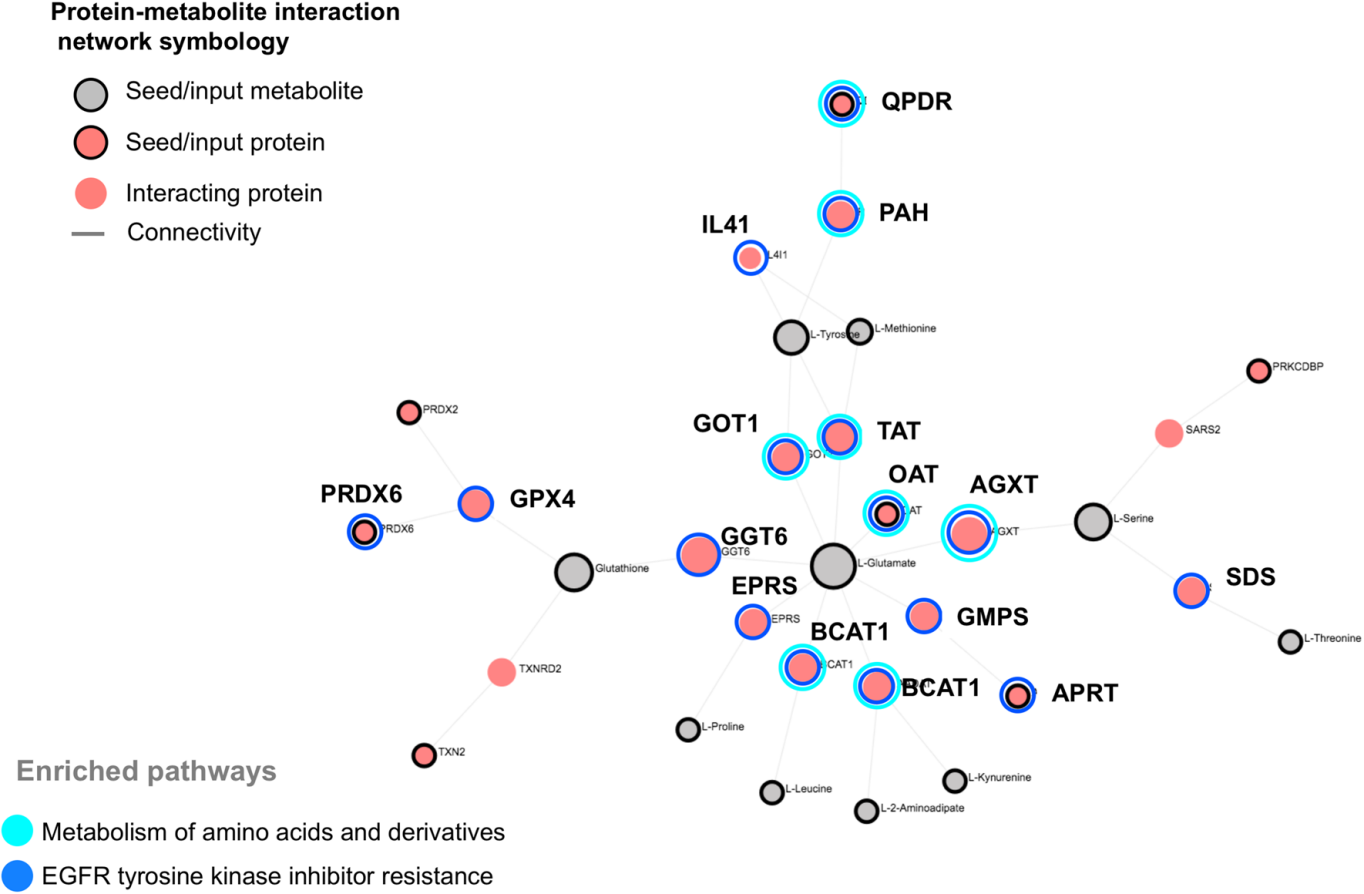
Integrative network of the proteomic and metabolomic perturbations caused by simulated diabetes in bovine coronary artery endothelial cells (BCAEC). Composite protein-metabolite network created by OmicsNet using dysregulated proteins (pink nodes with black shade) and metabolites (gray nodes) in the HG + HI group (simulated diabetes). Interacting proteins (< 0.9 confidence) were retrieved from STRING Database and are shown as pink nodes without black shade. Abbreviations: *NG* normal glucose, *HG* high glucose, *HI* high insulin.

The signaling pathways perturbed by simulated diabetes were identified by a composite network of interacting metabolites and proteins using OmicsNet built-in databases. Figure 6 illustrates the composite metabolite-PPI network generated, using the dysregulated molecules (under simulated diabetes), comprised of 10 metabolites (seed metabolites), 32 edges, and 21 proteins (7 seed proteins). The enriched signaling pathways identified in the composite network are shown in Table 2. Generally speaking, signaling pathways linked to the metabolism of amino acids were significantly enriched within the protein-metabolite network. The pathways containing more hits (proteins) are highlighted (bue tones) in Fig. 6. We also noted a smaller interaction between the Acyl-protein thioesterase 1 (LYPLA1,UniProtKB:Q3MHR0) protein and a phosphatidylcholine metabolite.

**Table 2:** Integrative pathway enrichment analysis of the dysregulated proteins and metabolites by simulated diabetes.

REACTOME database	Total	Hits	FDR	KEGG database	Total	Hits	FDR
Metabolism of amino acids and derivatives	190	8	2.8E−6	Phenylalanine, tyrosine and tryptophan biosynthesis	5	4	3.9E−8
Phenylalanine and tyrosine catabolism	9	3	5.3E−4	EGFR tyrosine kinase inhibitor resistance	1490	16	2.2E−7
Metabolism	1490	12	1.5E−3	Phenylalanine metabolism	17	4	6.1E−6
Abnormal metabolism in phenylketonuria	4	2	9.8E−3	Cysteine and methionine metabolism	49	5	6.7E−6
				ABC transporters	75	5	4.6E−6
				Antifolate resistance	18	3	5.2E−4
				Valine, leucine and isoleucine biosynthesis	4	2	1.5E−3
				Alanine, aspartate and glutamate metabolism	36	3	2.9E−3
				Tyrosine metabolism	36	3	2.9E−3

### Cellular morphology

**Figure 7:**
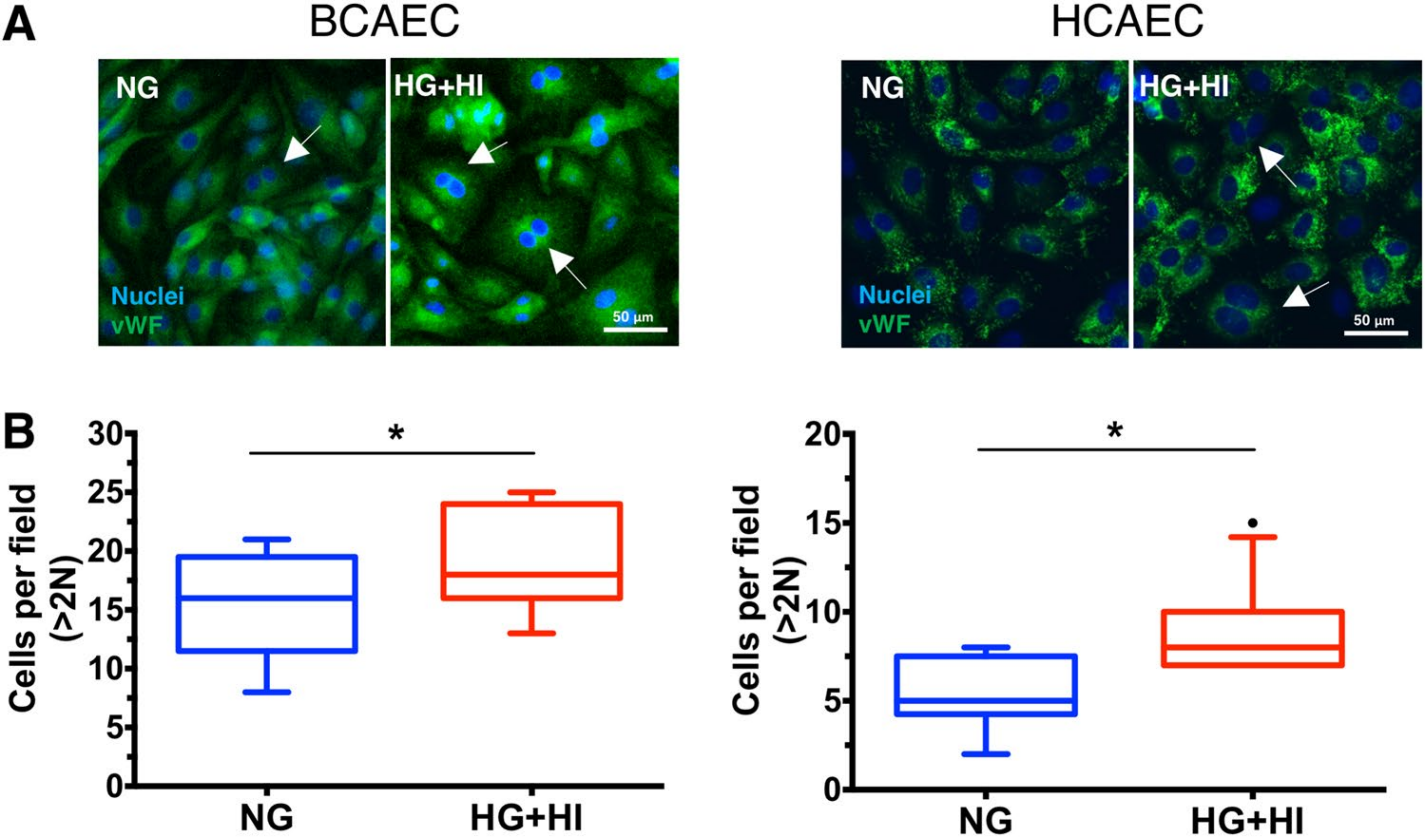
Increased cellular binucleation by simulated diabetes in bovine coronary artery endothelial cells (BCAEC) and human coronary artery endothelial cells (HCAEC). (**A**) Representative immunofluorescence micrographs showing the localization of the von-Willebrand factor (vWf, 1:400, 3% BSA in PBS) in fixed and permeabilized cells. The nuclei were stained using the dye Hoechst 33258 (2 µg/mL in HBSS). White arrows indicate binucleated cells. (**B**) Quantification of binucleated cells in HCAEC and BCAEC under simulated diabetes (HG + HI) versus control (NG) group. Fluorescence images were taken in at least three random fields per condition using an EVOS FLoid Cell Imaging Station with a fixed × 20 air objective. Image analysis was performed by ImageJ software (version 2.0.0). Abbreviations: *NG* normal glucose, *HG* high glucose, *HI* high insulin.

To better understand the effects that simulated diabetes exerts on endothelial cells the changes on cellular structure endpoints were evaluated. The endothelial nuclei morphology in the BCAEC control and experimental groups were evaluated using fluorescent-staining and image analysis. We also evaluated the presence of vWF (marker of endothelial cells) in BCAEC and HCAEC, to reveal the cellular boundary and to demonstrate their endothelial phenotype^47^. We noted an increase in the percentage of binucleated BCAEC in the experimental group compared to the control group (top panel Fig. 7A and B). A similar result with larger nuclei, was observed when using HCAEC as a human in vitro model (bottom panel Fig. 7A and B). Finally, as expected, we observed a typical intracellular localization of vWF and a 100% positivity in endothelial cells.

## Discussion

Endothelial cells cover the inner surface of blood vessels and are distributed across the body. Their functions include: acting as a mechanical barrier between the circulating blood and adjacent tissues as well as modulating multiple functions in distinct organs^48^. These regulatory functions vary according to localization and vascular bed-origin^49^. HG blood levels are detrimental to endothelial cells function in T2DM leading to coronary endothelial dysfunction and development of CVD^50,51^. The molecular effects of HG on endothelial cells have been previously characterized^4,6,7,10,11^; nevertheless, the endothelial cell types used in these studies are not intrinsically involved in CVD. The present study used an in-vitro model involving endothelial cells that modulate heart function, CAEC^52^. Noteworthy, most in-vitro published studies have arbitrarily selected a single concentration of HG and time point as a means to decipher the molecular mechanisms altered by simulated diabetes^4,6,7,10,11^. Before selecting a particular HG concentration, we first tested the effects of increasing concentrations of glucose with and without HI on the BCAEC mitochondria. The mitochondrial membrane potential was selected as an endpoint and surrogate marker for mitochondrial function and metabolism^19^, given that mitochondria are highly relevant for glucose metabolism and ATP production and because mitochondrial dysfunction is thought to contribute to T2DM^53^. Our approach consisted of pre-treating (before challenging them with HG) cells with HI for 3 days to create a prolonged hyperinsulinemic environment and then challenging cells with HG + HI for up to 9 days, trying to mimic the pathophysiological conditions that occur in T2DM subjects wherein hyperinsulinemia precedes hyperglycemia^18^. We noted a significant decrease in the mitochondrial membrane potential when using 20 mmol/L HG + 100 nmol/L HI until day 9 (Fig. S1), which is consistent with other reports using HG or HI on muscle cells^54^, endothelial cells^55^, epithelial cells^56^, and hepatocytes^57^. Mannitol, at equivalent concentrations on day 9, did not reduce the membrane potential; thus, we could rule out a hyperosmolar effect. We, therefore, for all our experiments, employed 20 mmol/L HG + 100 nmol/L HI for 9 days as the simulated diabetes model. Noteworthy, 9 days was the longest time point analyzed trying to mimic a chronic HG exposure and preventing measuring cell proliferation known to occur in early HG^10,12^.

Manual inspection of the proteomics data revealed dysregulated proteins involved in the signaling of insulin; a serine/threonine-protein phosphatase (PPP2R2B, [UniProtKB:Q5E9Q7]) and 14-3-3 adapter protein (YWHAB, [UniprotKB: P68250]). Elevated levels of the former protein, are linked to insulin resistance, reduced GLUT4 translocation and glucose transport by inhibition of AKT activity; a serine/threonine kinase responsible for the phosphorylation of the insulin receptor substrate (IRS-1, a direct intracellular effector of the insulin receptor) and mediator of down-stream insulin signaling^58^. On the other hand, 14-3-3 adapters are up-stream proteins essential in the signaling by insulin^59^. When insulin binds to its receptor, 14-3-3 interacts with the insulin receptor substrate 2 (IRS-2) and promotes the activation of PI3-kinase (activator of AKT) and subsequent down-stream signaling. Simulated diabetes augmented and reduced the protein levels of PPP2R2B and YWHAB in BCAEC, respectively, which suggest impaired insulin signaling in our model.

This LC–MS^2^-based methodological pipeline that included appropriate controls during data acquisition (QC) and processing (e.g., normalization, filtering, annotation, dereplication, etc.), allowed the identification of global changes in the metabolome and peptidome of CAEC under HG + HI. Using the GNPS/NAP pipeline we retrieved biological information at the metabolome level. Specifically, we noted increased abundance of valine, leucine, tyrosine, serine, leucine, proline, methionine, and glutamic acid in cells under HG conditions; and this is consistent with reports on human aortic endothelial cells^60^. Notably, several clinical studies have established a direct relationship between prevalence/incidence of T2DM and increased levels of valine, leucine and tyrosine in serum and plasma^61–65^. Our results support the role of CAEC in contributing to the elevated pool of amino acids seen in circulation under a HG environment. We speculate that increased levels of these amino acids could result from either increased production or reduced utilization as suggested in endothelial cells (immortalized cell line, EA.hy 926) that transition from a glycolytic metabolism towards lipid and amino acid oxidation when challenged by HG^66^. Furthermore, evidence of increased tryptophan catabolism was identified through the kynurenine pathway. In this regard, a non-significant decrease of ~ 40% in the abundance of tryptophan was detected. However, a significant increase of ~ 450% in kynurenine (tryptophan’s main metabolite)^67^ between the HG + HI group and NG group was also observed, which is a key finding as elevated plasma levels of kynurenine are known to increase CVD risk^68,69^. This novel finding contributes to expanding the understanding of amino acid metabolism in endothelial cells under simulated diabetes. Acetyl serotonin and melatonin which are components of the serotonin pathway that degrades tryptophan^70^ were also detected with only minor abundancy increases (20–30%) in the HG + HI group compared to control.

In endothelial cells, the tripeptide glutathione (cysteine-glutamic acid-glycine) is believed to be the most critical antioxidant thiol scavenging reactive oxygen species when oxidative insults (e.g., ambient HG) are triggered^71,72^. Here, we noted an increased abundance of glutathione and its metabolite precursor glutamic acid^71^, suggesting an increased response to oxidative stress by ambient HG and HI. In line with this evidence, previous research reported a glutathione-dependent reaction to ambient HG in artery-derived endothelial cells^73,74^ but the same could not be observed in vein-derived endothelial cells^75,76^. That emphasizes the different responses to HG among endothelial phenotypes. The use of exhaustive and complementary dereplication tools further allowed us to provide evidence of up-regulated novel glutathione-based metabolites, suggesting a coordinated boost of antioxidant metabolite peptides. Likewise, antioxidant enzymes conforming the cells natural enzymatic defense^77^ were found with increased abundance in the experimental group, including peroxiredoxin-2 (PRDX2, [UniProtKB:Q9BGI3], peroxiredoxin-6 [ PRDX6, UniProtKB:O77834]), and mitochondrial thioredoxin (TXN2, [UniProtKB:Q95108]). Our methodology also allowed us to identifying other peptide metabolites, including glutamic acid- and phenylalanine-based metabolites, presumably di- or tri-peptides, including the annotated metabolite glutamyl-phenylalanine and leucine-phenylalanine.

To further extract biological information at the peptidome level, we performed an in-silico analysis with PeakView software on the same LC–MS^2^ datasets used for untargeted metabolomics. GNPS/NAP and PeakView pipelines use MS^2^ spectral matching between experimental and reference spectral data for the identification of peptides, but they rely on different reference spectral databases. Regarding peptide metabolites, GNPS/NAP^26,27,30^ pipeline is focused principally on the identification of peptidic natural products (as well as other types of metabolites), while the Paragon algorithm in PeakView software^78^ allows identifying endogenous peptides (by in-silico prediction) derived from an organism’s proteome when used as a reference (e.g., human, animals). The CAEC peptidome analysis by PeakView pipeline suggested an increase in proline-containing peptides. This type of peptide is of particular interest because of its resistance to non-specific proteolytic degradation, body distribution and remarkable biological effects^79–82^. The origin of phenylalanine- and proline-based peptides may be derived from protein degradation supported by the noted increased protein abundance of core and regulatory subunits from the proteasome complex, including PSMA4 [UniProtKB:Q3ZCK9] and PSMD3 [UniProtKB:Q2KJ46]). This complex is a central proteolytic system that degrades proteins, releasing peptides with 3 to 22 residues for further degradation into amino acids^83^. Yet, the question to be answered is if these peptides are biologically active or merely products of protein degradation. However, that is beyond the scope of our study.

**Figure 8:**
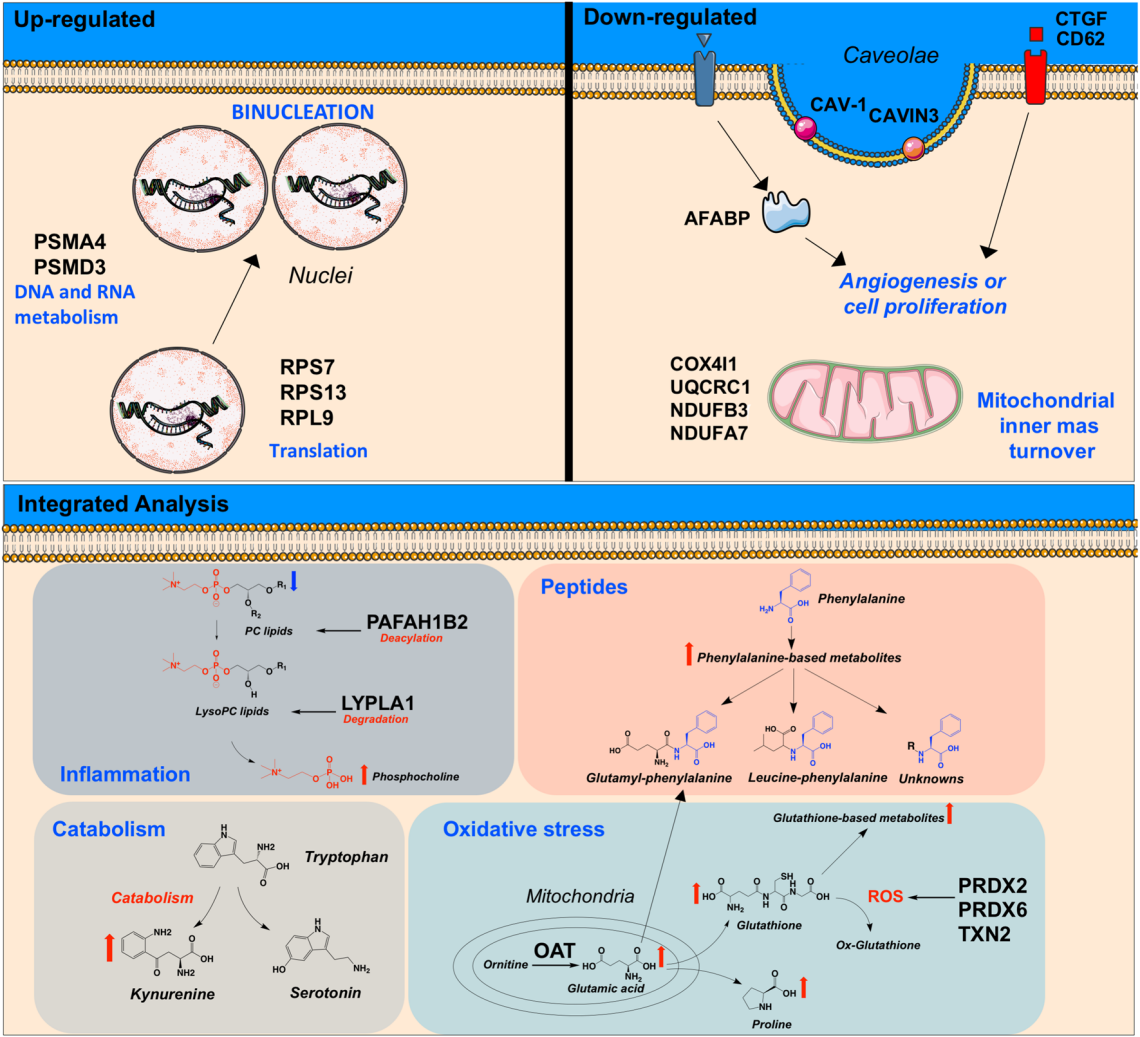
Summary illustration of study findings. Cellular structures were created using Servier Medical Art templates, which are licensed under a Creative Commons Attribution 3.0 Unported License; https://smart.servier.com. Chemical structures were drawn by ChemDraw Professional version 16.0.1.4.

Metabolomic profiling also revealed changes in the lipidome of CAEC challenged with HG + HI, wherein a reduction in phosphatidylcholine (PC) lipids and subsequent increase in phosphocholine were noted. Changes in the phospholipidomic profile of bovine aortic endothelial cells treated with HG for 24 h has been reported in a lipidome study^84^. Here, proteomics and metabolomics data were manually integrated and this allowed to determine critical roles for PAFAH1B2 (UniProtKB:P68401) and LYPLA1 in mediating the degradation of PC lipids (Fig. 8). PAFAH1B2 was found to be up-regulated in this study and it is known to be associated with inflammation and higher levels of lysoPC^85^. As a result, PAFAH1B2 could increase the pool of lysoPC lipids, further exacerbating inflammation in the cardiovascular system^86^. On the other hand, LYPLA1 has a lysophospholipase activity that can hydrolyze a range of lysophospholipids, including LysoPC, thereby generating a fatty acid and glycerophosphocholine as products^87^. Increased levels of phosphocholine (~ 460%) were detected in HG treated cells compared to control, that could be associated with the degradation of LysoPC lipids. It should be noted that the use of pathways databases such as KEGG and REACTOME possess some limitations when dealing with lipid metabolites because its chemical diversity is not well annotated/defined within the databases. For example, KEGG provides a chemical class identifier instead of individual identity to lipids, constricting their biological importance^88^. Thus, based on our manual inspection of the metabolomics-proteomics data and in line with the evidence, we suggest that simulated diabetes evokes inflammation on BCAEC and that PAFAH1B2 and LYPLA1 play a role in modulating such process.

Previously, we reported the multinucleation of CAEC cultured under simulated diabetes^16^. In the present study, we replicated our previous findings of increased binucleation in BCAEC. Notably, the same outcome was obtained when using HCAEC as a human in vitro model (Fig. 7A and B), validating the binucleation process in other CAEC, which indicates that such a process is not species specific but rather a true biological process. After refinement of LC–MS^2^ data and bioinformatics re-processing of published SWATH-based datasets of BCAEC under simulated diabetes^16^, molecular signatures and pathways that could be linked to the binucleation process were found (Fig. 8). For instance, we noted an increased abundance of proteins, under simulated diabetes, with reported nuclei localization and linked to DNA metabolism, including ribosomal proteins RPS7 (UniProtKB:A6H769), RPS13 (UniProtKB:Q56JX8), and RPL9 (UniProtKB:Q3SYR7)^89^. Further, we observed an increased abundance of proteasome proteins, PSMA4 and PSMD3, which are linked to protein metabolism^90^. Hence, we infer that the CAEC binucleation occurs as a compensatory mechanism to increase the cell capacity to metabolize the excess of ambient glucose by increasing the cell metabolic machinery (transcription/translation processes).

Despite a lack of apparent increase in cell proliferation in the experimental group compared to control group after 12 days, an increase in overall protein abundance was inferred from the total ion chromatogram (TIC) of MS (Fig. S1A). Previous studies have shown reduced endothelial cell proliferation (mostly in HUVEC) after long-term (7–14 days) HG exposure^4,11,91–97^, accompanied by an increase in protein synthesis^97^. Although an increase in cell proliferation could boost a coordinated increase of ribosomal and proteasome proteins, we do not believe this is the case here, as mentioned before. After 4–5 days of simulated diabetes, cells occupied 100% of the well's plate surface, thereby impeding to harbor more cells because endothelial cells grow as a monolayer. This is consistent with findings stating that when endothelial cells become highly confluent, they stop growing due to cell–cell contact, even in the presence of growth factors^98^. In support of this, up-stream (CTGF [UniProtKB:O18739]) and CD62 [UniProtKB:P98107])^99,100^ (Table S5) and down-stream proteins (FABP4 [UniProtKB:P48035])^101^ (Table S5) involved in angiogenesis and proliferation were down-regulated by simulated diabetes. Importantly, there is evidence (not in endothelial cells) of cellular processes contributing to the stimulation of cellular binucleation without increases in cell proliferation, including cellular enhancement of antimicrobial defenses^102^, senescence^103^, and malignancy^104^. Various mechanisms have been linked to the binucleation process, such as cytokinesis failure, cellular fusion, mitotic slippage, and endoreduplication^105^. The elucidation of the exact molecular mechanisms leading to the binucleation process of CAEC is beyond the scope of our study.

The chosen period (9-days) to simulate diabetes may represent a limitation, as longer periods may better reflect chronicity and trigger more severe endothelial dysfunction. The findings observed in BCAEC may not be equivalent to those occurring in human cells. However, the binucletation process triggered in BCAEC by simulated diabetes was also observed in human endothelial cells.

In conclusion, integration of omics and bio/chemoinformatics data revealed dysregulations in the metabolism of amino acids, peptides, and phospholipids, impaired insulin signaling, reduced mitochondrial mass, angiogenesis, and increased apoptosis and oxidative stress when CAEC were subjected to simulated diabetes. The appearance of non-proliferative binucleated CAEC cells is thought to be a strategy to metabolize the excess ambient glucose was also reported. Collectively, we believe that these dysregulated factors contribute to the development of CAEC dysfunction and may be associated with critical mechanisms underlying the onset of CVD in subjects with T2DM. We, therefore, suggest a multi-target therapeutic modality when protecting diabetic patients from CVD.

## Supplementary Information

Supplementary Information.

Supplementary Table S1.

Supplementary Table S3.

Supplementary Table S5.

## Data Availability

The raw datasets supporting the metabolomics results are available in the GNPS/MassIVE public repository^27^ under the accession number MSV000084307. The specific parameters of the tools employed for metabolite annotation are available on the following links: for classical molecular networking, https://gnps.ucsd.edu/ProteoSAFe/status.jsp?task=604b3d077e00430a9bc288eebf154b9b; for FBMN https://gnps.ucsd.edu/ProteoSAFe/status.jsp?task=5e2839037969442e868d9df21309d561; for NAP, https://proteomics2.ucsd.edu/ProteoSAFe/status.jsp?task=96cda48c0df64d3398a8f9088907afb5; for MS2LDA, http://ms2lda.org/basicviz/summary/1197/ (need to log-in as a registered or guest user); for MolNetEnhancer, https://gnps.ucsd.edu/ProteoSAFe/status.jsp?task=de80b9c765e042ffab7767a3101054fd. The quantitative results generated using the XCMS platform can be accessed after logging into the following link https://xcmsonline.scripps.edu and searching for the job number 1395724. SWATH data is accessible on the ProteomeXchange with dataset identifier PXD013643.
