# Supplementary material for: Multi-omics study identifies novel signatures of DNA/RNA, amino acid, peptide, and lipid metabolism by simulated diabetes on coronary endothelial cells
Source: Sci Rep. 2022 Jul 14;12:12027. doi: 10.1038/s41598-022-16300-5 (PMC9283518; doi:10.1038/s41598-022-16300-5)
Supplement: Supplementary file 1 — Supplementary Information. [file 41598_2022_16300_MOESM1_ESM.docx]

**SUPPORTING INFORMATION**

**Multi-omics study identifies novel signatures of DNA/RNA, amino acid, peptide, and lipid metabolism by simulated diabetes on coronary endothelial cells**

**Aldo Moreno-Ulloa**^1,2^***, Hilda Carolina Delgado-De la Herrán**^1,3,4^**, Carolina Álvarez-Delgado**^4^**, Omar Mendoza-Porras**^5^**, Rommel A. Carballo-Castañeda**^1,3^, **Luis Donis-Maturano**^6^, **and Francisco Villarreal**^7,8^

^1^MS2 laboratory, Biomedical Innovation Department, CICESE, Baja California, México

^2^Specialized Laboratory in Metabolomics and Proteomics (MetPro), CICESE, México

^3^Posgrado en Ciencias de la Vida, CICESE, Baja California, México

^4^Mitochondrial Biology Laboratory, Biomedical Innovation Department, CICESE, Baja California, México

^5^CSIRO Livestock and Aquaculture, Queensland Bioscience Precinct, 306 Carmody Rd, St Lucia, QLD, Australia

^6^Unidad de Investigación en Biomedicina (UBIMED), Facultad de Estudios Superiores (FES)-Iztacala, UNAM, Estado de México, México

^7^School of Medicine, University of California, San Diego, CA, USA

^8^San Diego VA Healthcare System

* To whom correspondence should be addressed: Biomedical Innovation Department, CICESE Carretera Ensenada-Tijuana No. 3918, Zona Playitas, CP. 22860, Ensenada, B.C. Mexico, Phone: +52(646)175-05-00 ext. 2721, E-mail: [amoreno@cicese.mx](mailto:amoreno@cicese.mx)

**This PDF file includes:**

Supplemental Experimental Methods

Supplemental Figures 1-3:

Supplemental Tables S2 and S4

Supplemental References

**Figure S1. Time- and concentration-dependent effects of glucose and insulin on mitochondrial function.** Bovine Coronary Artery Endothelial Cells (BCAEC) were incubated for up to 9 days with normal-glucose (NG, 5.5 mM) or high-glucose (HG, 15, 17.2, or 20 mM) with and without 100 nM insulin (high-insulin, HI). As hyperosmolar controls, cells were incubated with 5.5 mmol/L glucose + mannitol 9.5 (High-mannitol, 15 mM), 12.2 (High-mannitol, 17.2 mM) or 14.5 mmol/L (High-mannitol, 20 mM) with or without HI for 9 days. As a surrogate marker of mitochondrial function, mitochondrial membrane potential in viable non-fixed cells was evaluated by MitoTracker Red FM (100 nM for 30 min) and flow cytometry. Values were normalized (set to 1) to the averaged levels of the NG group of each condition. **p-value* <0.05 vs. NG (same condition), ^NS^*p-value* >0.05 vs. NG (same condition) (One-way ANOVA, post hoc test Dunnett’s),^ *p-value* <0.05 vs. same condition with HM (unpaired, student’s t test). Results are expressed as mean and standard error of the mean. All experiments were carried out in triplicate. Abbreviations: HM, high-mannitol; MFI, median fluorescence intensity; MTR FM, MitoTracker Red FM; NS, non-significant.

**Figure S2. Proteomics data normalization results using NormalyzerDE**. (A) Total intensity of raw data before normalization. (B) Quantitative parameters of normalization algorithms (pooled intragroup coefficient of variation [PCV], median absolute deviation [PMAD], estimate of variance [PEV]). Qualitative parameters of normalization algorithms; (C) Box plots (D) MA plots, and (E) Density plots.

**Figure S3. Cellular confluence in control and experimental group.** Representative micrographs of Bovine Coronary Artery Endothelial Cells (BCAEC) cultured for 9 days with 5.5 mmol/L glucose (control group) and 20 mmol/L glucose+100 nmol/L insulin (simulated diabetes or experimental group). Images were taken using an EVOS FLoid Cell Imaging Station with a fixed 20x air objective. Abbreviations: NG, normal glucose; HG, high glucose; HI, high insulin.

**Table S2.** List of the putatively annotated metabolites modulated by simulated diabetes using GNPS spectral matching and *CSI:FingerID* prediction.

**Table S4.** List of the putatively annotated proline-peptides altered by simulated diabetes using ProteinPilot Software and manual inspection.

In addition, this manuscript also has associated 3 .xlsx files corresponding to **Table S1, S3, and S5**.

**Table S1.** List of all the putatively annotated metabolites by MS^2^ spectral matching against GNPS public spectral libraries.

**Table S3.** List of all detected peptides by ProteinPilot Software using the metabolomics datasets.

**Table S5.** List of the detected peptides and proteins in all conditions for SWATH-based quantification.

**SUPPLEMENTAL EXPERIMENTAL METHODS**

**Metabolomics data processing.** For the XCMS pipeline or approach 1, raw LC-MS^2^ data files (.wiff and .wiff scan) were uploaded to the XCMS web-based platform at <https://xcmsonline.scripps.edu> ^1^. Preset parameters for TripleTOF 5600 in positive ionization mode and pairwise (unpaired t test) were selected, with the following modifications; min peak width, 15 s; max peak width, 90 s; feature detection mass tolerance, 15 ppm; and peak normalization, median fold change. For the MZmine pipeline or approach 2, raw data files (.wiff and .wiff scan format) were first converted to .mzML using MSconvert (ProteoWizard)^2^. Mass detection, chromatogram building and deconvolution (ADAP algorithm), isotopic assignment, feature alignment and gap-filling (to detect features missed during the initial alignment) were performed in MZmine Version 2.38^3^. Features with at least 2 isotopes were kept and the list containing all MS^1^ was exported as a .csv file. The list comprising all the features detected by MZmine was used for comparison against the XCMS pipeline. For subsequent analysis, features detected in mobile phase (water/acetonitrile 95:5 v/v with 0.1% formic) blanks were considered contaminants and removed from samples using the *peak list row filter* tool within MZmine and manual inspection. Features with MS^2^ data were kept. The peak areas and MS^2^ data of filtered features were exported as .csv and .mgf files, respectively. This was followed by Feature Based Molecular Networking (FBMN) and spectral library matching performed into the Global Natural Products Social Molecular Networking (GNPS) web-platform ([www.gnps.ucsd.edu)](http://www.gnps.ucsd.edu)) ^4^. The parameters used within the GNPS were precursor ion and product ion mass tolerance of 0.02 Da. The molecular network was created using a minimum cosine score of 0.6 and a minimum of 4 peaks matched. The spectra in the network were searched against GNPS spectral libraries using a minimum cosine score of 0.6 and at least 4 matched peaks as filters. To expand the annotation of the metabolites not automatically retrieved by spectral matching, the GNPS *in silico* tool, Network Annotation Propagation (NAP), was used ^5^. NAP uses the output of molecular networking to rerank *in silico* candidate structure lists, which has been shown to improve metabolite annotation. For NAP, adducts [M+NH_4_]+, [M+H]+, [M+Na]+, and [M+K]+ with *m/z* tolerance set to 10 ppm were searched using the parameters described at: <https://proteomics2.ucsd.edu/ProteoSAFe/status.jsp?task=96cda48c0df64d3398a8f9088907afb5>. A chemical class was automatically assigned to all putative annotations obtained through GNPS library matching and NAP, using the Classyfire chemical ontology nomenclature^6^. Molecular networking, NAP, and Classyfire outputs were integrated using the MolNetEnhancer workflow to visualize the metabolome detected^7^. As a complementary step to annotate metabolites, chemical substructures were recognized using the MS2LDA computational tool aiming to discover co-occurring fragments and neutral losses (referred as to Mass2Motifs [M2M]) within the MS2 data (using the same .mgf file as in GNPS). This provides chemical information at the substructure level of the metabolites ^8^. MS^1^ quantification (with normalization “on”) was utilized to visualize relevant sub structurally-related features in MS2LDA. Fragments retrieved by M2M of interest were searched against the mzCloud database (<https://www.mzcloud.org)> to obtain Heuristic and Quantum Chemical predictions (non-trivial *in silico* predictions). To compare the features detected by both platforms, the lists of MS^1^ were transformed to mzTab format and imported into MZmine for feature alignment using a tolerance of 0.01 m/z and 1 min. The aligned features were considered the same entity. A Venn diagram was used to visualize and compare the features detected by MZmine and XCMS software. For select dysregulated metabolites by simulated diabetes, the ZODIAC tool ^9^ integrated in SIRIUS software was utilized for molecular formula assignment, while the CSI:FingerID tool ^10^ also integrated in SIRIUS was utilized to retrieve high-confidence molecular structures. As a complementary approach, the chemical classes of the dysregulated metabolites were determined by the class assignment and ontology prediction using mass spectrometry (CANOPUS) ^11^ computational tool in SIRIUS software. To speed up running times, we selected only compounds <860 Da. For approach 3, normalized peak or feature abundance data retrieved by XCMS was imported (.txt format) into the Statistical Analysis Module of Metaboanalyst 5.0^12^. To improve the quantification precision, data was filtered using the “Filtering features if their (RSD) are> 25% in QC samples” option.

**REFERENCES**

1 Gowda, H. *et al.* Interactive XCMS Online: simplifying advanced metabolomic data processing and subsequent statistical analyses. *Anal Chem* **86**, 6931-6939, doi:10.1021/ac500734c (2014).

2 Holman, J. D., Tabb, D. L. & Mallick, P. Employing ProteoWizard to Convert Raw Mass Spectrometry Data. *Curr Protoc Bioinformatics* **46**, 13 24 11-19, doi:10.1002/0471250953.bi1324s46 (2014).

3 Pluskal, T., Castillo, S., Villar-Briones, A. & Oresic, M. MZmine 2: modular framework for processing, visualizing, and analyzing mass spectrometry-based molecular profile data. *BMC Bioinformatics* **11**, 395, doi:10.1186/1471-2105-11-395 (2010).

4 Nothias, L. F. *et al.* Feature-based molecular networking in the GNPS analysis environment. *Nat Methods* **17**, 905-908, doi:10.1038/s41592-020-0933-6 (2020).

5 da Silva, R. R. *et al.* Propagating annotations of molecular networks using in silico fragmentation. *PLoS Comput Biol* **14**, e1006089, doi:10.1371/journal.pcbi.1006089 (2018).

6 Djoumbou Feunang, Y. *et al.* ClassyFire: automated chemical classification with a comprehensive, computable taxonomy. *J Cheminform* **8**, 61, doi:10.1186/s13321-016-0174-y (2016).

7 Ernst, M. *et al.* MolNetEnhancer: Enhanced Molecular Networks by Integrating Metabolome Mining and Annotation Tools. *Metabolites* **9**, doi:10.3390/metabo9070144 (2019).

8 van der Hooft, J. J., Wandy, J., Barrett, M. P., Burgess, K. E. & Rogers, S. Topic modeling for untargeted substructure exploration in metabolomics. *Proc Natl Acad Sci U S A* **113**, 13738-13743, doi:10.1073/pnas.1608041113 (2016).

9 Marcus Ludwig, L.-F. N., Kai Duhrkop, Irina Koester, Markus Fleischauer, Martin A. Hoffmann, Daniel Petras, Fernando Vargas, Mustafa Morsy, Lihini Aluwihare, Pieter C. Dorrestein, Sebastian Bocker. Database-independent molecular formula annotation using Gibbs sampling through ZODIAC. *Nature Machine Intelligence* **2**, 13, doi:<https://doi.org/10.1038/s42256-020-00234-6> (2020).

10 Duhrkop, K., Shen, H., Meusel, M., Rousu, J. & Bocker, S. Searching molecular structure databases with tandem mass spectra using CSI:FingerID. *Proc Natl Acad Sci U S A* **112**, 12580-12585, doi:10.1073/pnas.1509788112 (2015).

11 Duhrkop, K. *et al.* Systematic classification of unknown metabolites using high-resolution fragmentation mass spectra. *Nat Biotechnol* **39**, 462-471, doi:10.1038/s41587-020-0740-8 (2021).

12 Chong, J., Wishart, D. S. & Xia, J. Using MetaboAnalyst 4.0 for Comprehensive and Integrative Metabolomics Data Analysis. *Curr Protoc Bioinformatics* **68**, e86, doi:10.1002/cpbi.86 (2019).

**SUPPLEMENTAL FIGURES**

**
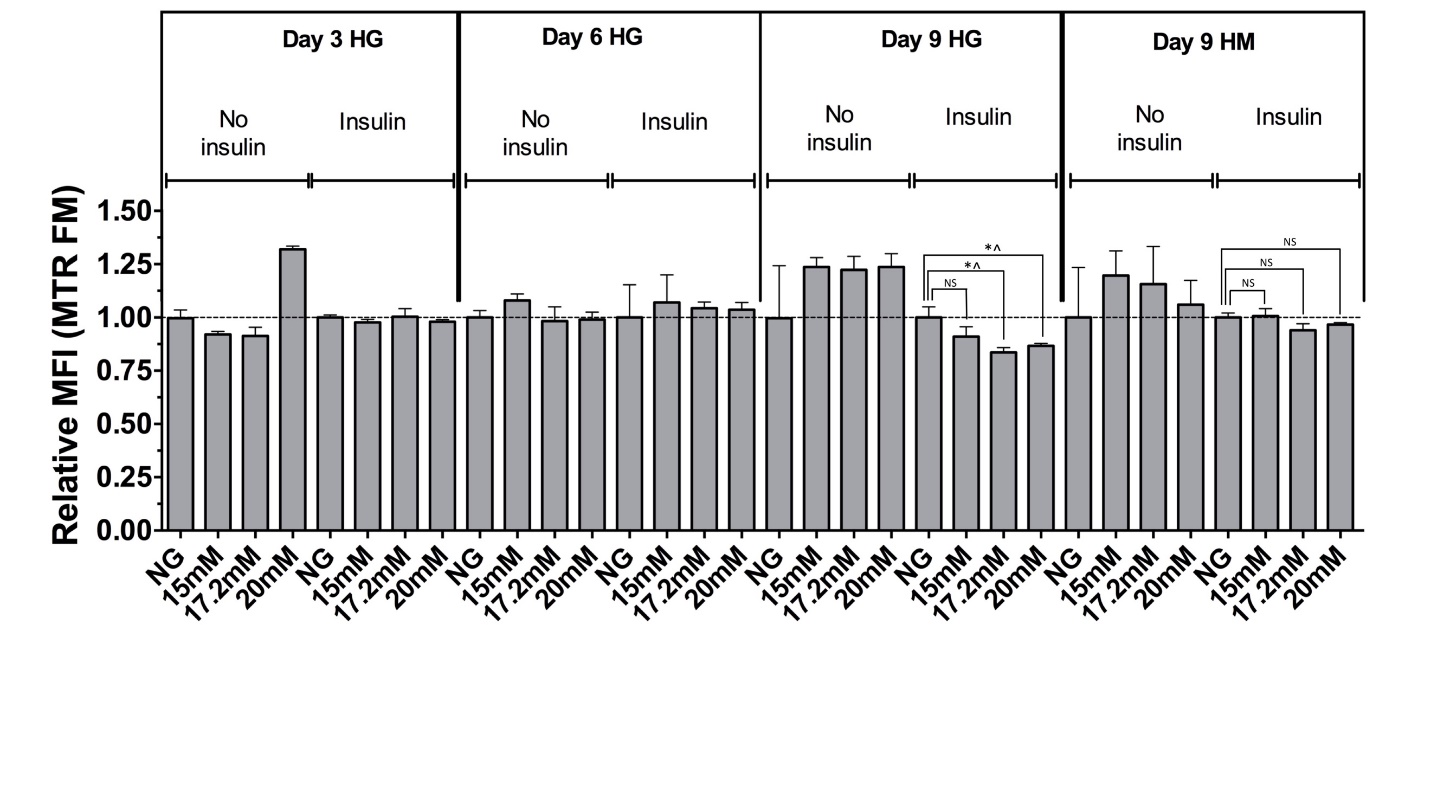
**

**Figure S1. Time- and concentration-dependent effects of glucose and insulin on mitochondrial function.** Bovine Coronary Artery Endothelial Cells (BCAEC) were incubated for up to 9 days with normal-glucose (NG, 5.5 mM) or high-glucose (HG, 15, 17.2, or 20 mM) with and without 100 nM insulin (high-insulin, HI). As hyperosmolar controls, cells were incubated with 5.5 mmol/L glucose + mannitol 9.5 (High-mannitol, 15 mM), 12.2 (High-mannitol, 17.2 mM) or 14.5 mmol/L (High-mannitol, 20 mM) with or without HI for 9 days. As a surrogate marker of mitochondrial function, mitochondrial membrane potential in viable non-fixed cells was evaluated by MitoTracker Red FM (100 nM for 30 min) and flow cytometry. Values were normalized (set to 1) to the averaged levels of the NG group of each condition. **p-value* <0.05 vs. NG (same condition), ^NS^*p-value* >0.05 vs. NG (same condition) (One-way ANOVA, post hoc test Dunnett’s),^ *p-value* <0.05 vs. same condition with HM (unpaired, student’s t test). Results are expressed as mean and standard error of the mean. All experiments were carried out in triplicate. Abbreviations: HM, high-mannitol; MFI, median fluorescence intensity; MTR FM, MitoTracker Red FM; NS, non-significant.

**
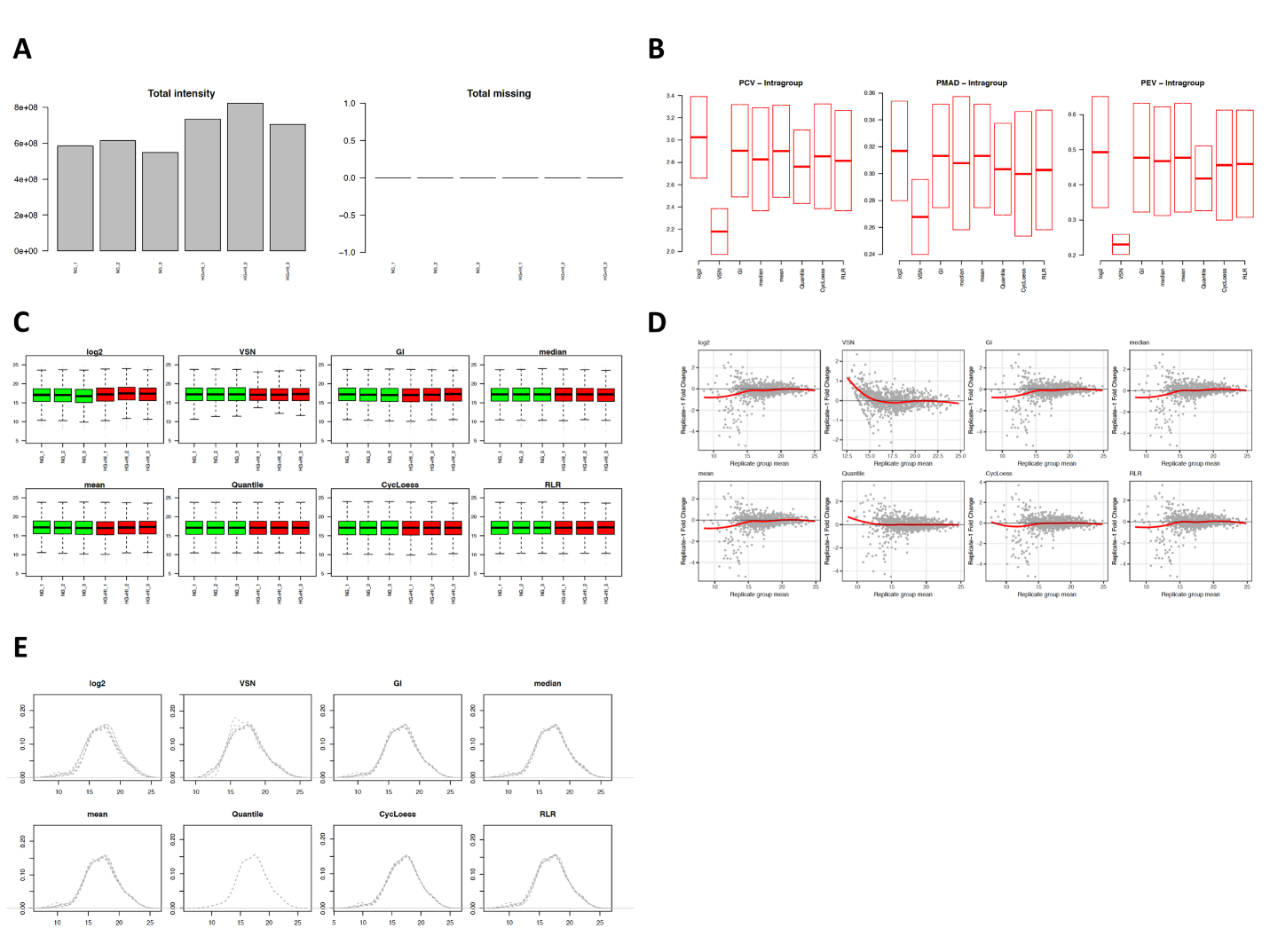
**

**Figure S2*.*** Proteomics data normalization results using NormalyzerDE. (A) Total intensity of raw LC-MS^2^ data before normalization. (B) Quantitative parameters of normalization algorithms (pooled intragroup coefficient of variation [PCV], median absolute deviation [PMAD], estimate of variance [PEV]). Qualitative parameters of normalization algorithms; (C) Box plots (D) MA plots, and (E) Density plots.

**
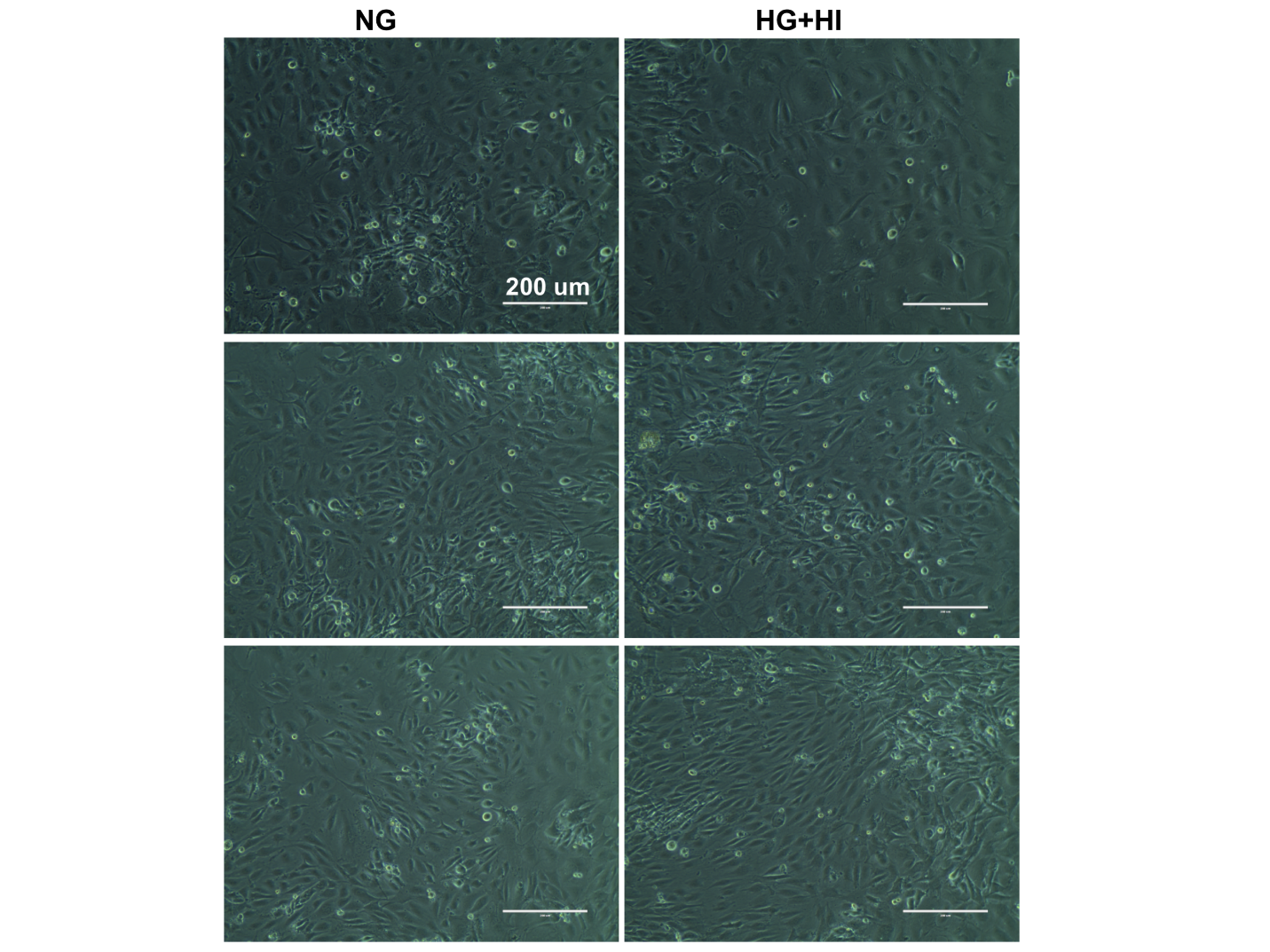
**

**Figure S3. Cellular confluence in control and experimental group.** Representative micrographs of Bovine Coronary Artery Endothelial Cells (BCAEC) cultured for 9 days with 5.5 mmol/L glucose (control group) and 20 mmol/L glucose+100 nmol/L insulin (simulated diabetes or experimental group). Images were taken using an EVOS FLoid Cell Imaging Station with a fixed 20x air objective. Abbreviations: NG, normal glucose; HG, high glucose; HI, high insulin.

**SUPPLEMENTAL TABLES**

**Table S2. List of the putatively annotated metabolites modulated by simulated diabetes using GNPS spectral matching and CSI:FingerID prediction.**

|  | **Compound**  **name** | **Chemical**  **subclass^a^** | **MSI**  **level^b^** | **RT**  **(min)** | **Experimental mass**  **[adduct]** | **Exact mass** | **Mass error**  **(ppm)** | **FC**  **(vs.ctrl)^c^** |
| --- | --- | --- | --- | --- | --- | --- | --- | --- |
| **Up-regulated** | Kynurenine | Carbonyl compounds | 2 | 3.2 | 209.0914  [M+H]^+^ | 209.0926 | -5.7 | 4.2 |
|  | Panthothenic acid | Alcohols and polyols | 2 | 2.8 | 242.0992  [M+Na]^+^ | 242.1004 | -4.9 | 1.38 |
|  | Leucine-phenylalanine | Aminoacids, peptides and analogues | 3 | 1.4 | 297.1802  [M+NH_4_]^+^ | 297.1814 | -4.0 | 1.99 |
|  | Asparaginyl- Asparaginyl-prolyl-serine | Aminoacids, peptides and analogues | 3 | 1.4 | 453.1704  [M+Na]^+^ | 453.1709 | -1.1 | 1.9 |
|  | Alanyl-valyl-aspartyl-proline | Aminoacids, peptides and analogues | 3 | 8.1 | 401.2028  [M+H]^+^ | 401.203 | -0.49 | 1.75 |
|  | Threonine | Aminoacids, peptides and analogues | 2 | 0.6 | 120.0658  [M+H]^+^ | 120.0661 | -2.4 | 1.4 |
|  | Valine | Aminoacids, peptides and analogues | 2 | 0.75 | 118.086  [M+H]^+^ | 118.0868 | -6.7 | 1.5 |
|  | Proline | Aminoacids, peptides and analogues | 2 | 0.6 | 116.0710  [M+H]^+^ | 116.0712 | -1.7 | 1.7 |
|  | Leucine | Aminoacids, peptides and analogues | 2 | 1.3 | 132.1022  [M+H]^+^ | 132.1025 | -2.2 | 1.37 |
|  | Serine | Aminoacids, peptides and analogues | 2 | 0.6 | 106.0504  [M+H]^+^ | 106.0504 | 0 | 1.5 |
|  | Glutamic acid | Aminoacids, peptides and analogues | 2 | 0.6 | 148.0604  [M+H]^+^ | 148.061 | -4.0 | 1.6 |
|  | Methionine | Aminoacids, peptides and analogues | 2 | 0.9 | 150.0584  [M+H]^+^ | 150.0589 | -3.3 | 1.4 |
|  | Tyrosine | Aminoacids, peptides and analogues | 2 | 1.3 | 182.0810  [M+H]^+^ | 182.0817 | -3.8 | 1.3 |
|  | Glutathione | Aminoacids, peptides and analogues | 2 | 1.3 | 615.1719  [2M+H]^+^ | 615.1755 | -5.8 | 2.1 |
|  | Glutamyl-phenylalanine | Aminoacids, peptides and analogues | 2 | 9.4 | 295.1285  [M+H]^+^ | 295.1294 | -3.0 | 1.4 |
|  | 2-aminoadipate | Aminoacids, peptides and analogues | 2 | 0.6 | 162.076  [M+H]^+^ | 162.0767 | -4.3 | 1.4 |
|  | 2,4-dimethylbenzoic acid | Benzoic acids and derivatives | 2 | 15.4 | 151.0753  [M+H]^+^ | 151.0759 | -3.9 | 1.4 |
|  | Phosphocholine | Quaternary ammonium salts | 2 | 0.75 | 184.0730  [M]^+^ | 184.0738 | -4.3 | 1.8 |
| **Down-regulated** |  |  |  |  |  |  |  |  |
|  | PC(18:1(9Z)/18:1(9Z))^d^ | Glycerophosphocholines | 2 | 24.4 | 786.5972  [M+H]^+^ | 786.6013 | -5.2 | -5.8 |
|  | PC(16:0/18:1(9Z))^d^ | Glycerophosphocholines | 2 | 24.8 | 760.5816  [M+H]^+^ | 760.5857 | -5.3 | -21.6 |
|  | (2S)-2-(6-Hydroxy-6-methyloctyl)-2H-furan-5-one | Furanones | 2 | 15.4 | 244.1906  [M+NH_4_]^+^ | 244.1912 | -2.4 | -1.86 |

^a^ Classification by Classyfire <http://classyfire.wishartlab.com>

^b^ Metabolomics Standards Initiative (MSI) quality level. Level 3, corresponds to CSI:FingerID high-confidence candidates (COSMIC Score >0.6). Level 2, corresponds to automatic spectral matching against GNPS spectral libraries.

^c^ Fold change of HG+HI/NG

^d^ The exact position and the cis/trans configuration could not be determined.

^ox^oxidized residue

^a^ Gene ID of the corresponding protein contained the peptide sequence

^b^high-glucose+high insulin_AUC_/normal glucose_AUC_

^c^Confidence threshold by ProteinPilot algorithm

| **Sequence** | **Gene^a^** | **Experimental mass**  **[adduct]** | **Exact mass** | **Mass error**  **(ppm)** | **Retention Time (min)** | **FC**  **(vs. control)^b^** | **p-value** | **Confidence**  **(%)^c^** |
| --- | --- | --- | --- | --- | --- | --- | --- | --- |
| TAPEIAVP | UFC1 | 399.2232  [M+2H]^+^ | 399.2238  [M+2H]^+^ | -1.5 | 9.4 | 1.46 | 0.04 | 99 |
| PPPPVP^ox^PPPPPP | WASF1 | 601.3339  [M+2H]^+^ | 601.3344  [M+2H]^+^ | -0.8 | 9.9 | 1.27 | 0.03 | 94.7 |
| LPP | Unknown | 326.2067  [M+H]^+^ | 326.2074  [M+H]^+^ | -2.2 | 8.4 | 1.45 | 0.01 | Manual inspection |

**Table S4.** **List of the putatively annotated proline-peptides altered by simulated diabetes using ProteinPilot Software and manual inspection**
